# New Carboxamides and a New Polyketide from the Sponge-Derived Fungus *Arthrinium* sp. SCSIO 41421

**DOI:** 10.3390/md20080475

**Published:** 2022-07-25

**Authors:** Jianglian She, Yi Chen, Yuxiu Ye, Xiuping Lin, Bin Yang, Jiao Xiao, Yonghong Liu, Xuefeng Zhou

**Affiliations:** 1 CAS Key Laboratory of Tropical Marine Bio-Resources and Ecology, Guangdong Key Laboratory of Marine Materia Medica, South China Sea Institute of Oceanology, Chinese Academy of Sciences, Guangzhou 510301, China; shejianglian20@mails.ucas.ac.cn (J.S.); 13620281931@163.com (Y.C.); xiupinglin@scsio.ac.cn (X.L.); yangbin@scsio.ac.cn (B.Y.); yonghongliu@scsio.ac.cn (Y.L.); 2 University of Chinese Academy of Sciences, Beijing 100049, China; 3 Southern Marine Science and Engineering Guangdong Laboratory (Guangzhou), Guangzhou 511458, China; 4 Institute of Marine Drugs, Guangxi University of Chinese Medicine, Nanning 530200, China; 18877548173@163.com; 5 Wuya College of Innovation, Shenyang Pharmaceutical University, Shenyang 110016, China; xj110121@126.com

**Keywords:** sponge-derived fungus, *Arthrinium* sp., carboxamides, acetylcholinesterase

## Abstract

New carboxamides, (±)-vochysiamide C (**1**) and (+)-vochysiamide B (**2**), and a new polyketide, 4*S*,3a*S*,9a*R*-3a,9a-deoxy-3a hydroxy-1-dehydroxyarthrinone (**3**), were isolated and identified from the sponge-derived fungus *Arthrinium* sp. SCSIO 41421, together with other fifteen known natural products (**4**–**18**). Their structures including absolute configurations were determined by detailed NMR, MS spectroscopic analyses, calculated electronic circular dichroism (ECD), as well as quantum-chemical NMR calculations. Preliminary bioactivity screening and molecular docking analysis revealed that several natural products exhibited obvious enzyme inhibitory activities against acetylcholinesterase (AChE), such as 2,3,6,8-tetrahydroxy-1-methylxanthone (**4**) with an inhibitory rate 86% at 50 μg/mL.

## 1. Introduction

Marine sponge derived fungi featured with easy culture and efficient productivity have been proven to be a prolific source of structurally diverse and novelty secondary metabolites [1]. Plenty of new secondary metabolites of sponge-derived fungi have been discovered with striking bioactive properties such as antifungal [2], antiviral [3], antioxidant [4], and cytotoxic [5] properties; meanwhile, their structures are diverse including polyketones, terpenes, and alkaloids [6,7]. 

*Arthrinium* sp. distributing in both terrestrial and marine habitats and comprising more than 32 species [8] produce a vast array of secondary metabolites, especially for xanthones, peptides, and terpenes [9], which showed a variety of activities covering antibacterial [9], cytotoxic [10], and antitumoral [11] activities.

**Figure 1:**
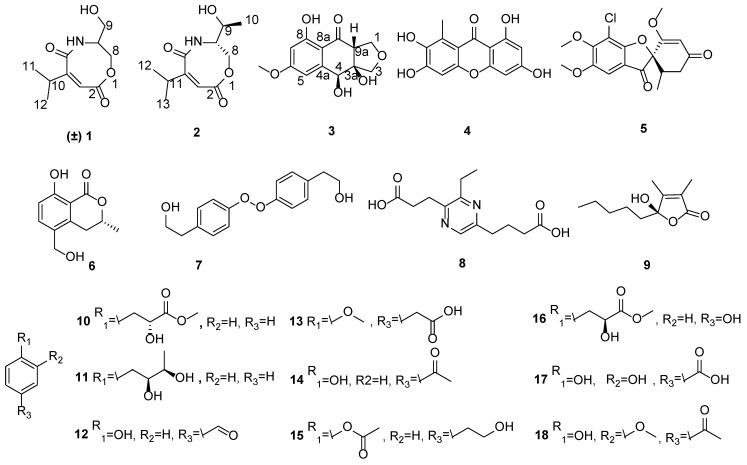
The structures of **1**–**18**.

In our research on bioactive natural products from marine sponge-derived fungi, two new carboxamides, (±)-vochysiamide C (**1**) and (+)-vochysiamide B (**2**), and one new polyketide, 4*S*,3a*S*,9a*R*-3a,9a-deoxy-3a hydroxy-1-dehydroxyarthrinone (**3**), were isolated and identified from the sponge-derived fungus *Arthrinium* sp. SCSIO 41421, together with fifteen known natural products (**4**–**18**) (Figure 1). Herein, we performed fermentation, purification, identification, and bioassay of **1**–**18**.

## 2. Results and Discussion

**Figure 2:**
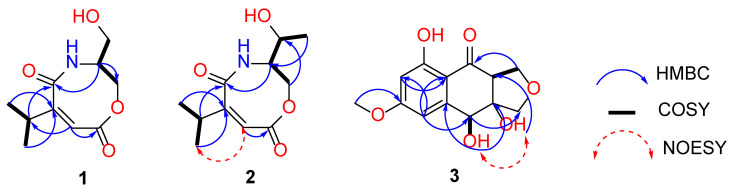
Key COSY, HMBC, and NOESY correlations of **1**–**3**.

Compound **1** was obtained as a colourless oil, and had the molecular formula C_10_H_15_NO_4_ (four degrees of unsaturation) as determined by its HRESIMS data, which showed a protonated ion peak at *m/z* 214.1077 ([M + H]^+^). The ^1^H and ^13^C NMR data (Table 1) showed one aromatic/olefinic methine (*δ*_C/H_ 124.9/6.53, C-3), two methyls (*δ*_C/H_ 20.6/1.15, C-11; 20.6/1.15, C-12), two methylenes (*δ*_C/H_ 58.3/3.56, C-8; 58.3/3.56, C-9), two methines (*δ*_C/H_ 25.1/2.71, C-10; 56.0/3.99, C-7), two carbonyls (*δ*_C_ 171.4, C-2; *δ*_C_ 171.2, C-5), and one hydroxy (*δ*_H_ 4.79). Based on the detailed analysis of its HMBC spectrum (Figure 2), which exhibited correlations from H-3 to C-4 and C-2, from H-8 to C-7, from H-9 to C-7, and from H-10 to C11, C12, C-3, C-4, and C-5, a ring containing carboxamide was deduced. The configuration of *∆*^3^ was established as *Z* through NOESY correlations observed between the isopropyl group and H-3 (Figure 2). Although 1 has one stereogenic carbon (C-7), its optical rotation was close to zero and the inapparent Cotton effect in the electronic circular dichroism (ECD) spectra suggested that **1** was not enantiomerically pure. Unfortunately, with many attempts for various chiral columns and mobile phase systems, **1** could not be successfully separated and was named as a racemic (±)-vochysiamide C.

Compound **2** was obtained as a white solid and was determined to have the molecular formula C_11_H_17_NO_4_ from the HRESIMS data, a protonated ion peak at *m/z* 228.1233 ([M + H]^+^). Analysis of the ^1^H and ^13^C NMR data (Table 1) of 2 with those of 1 displayed almost superimposable structural relationships. The remarkable distinction was the replacement of a hydrogen at C-9 in 1 with a methyl (*δ*_C/H_ 20.9/1.19, C-10) in 2. The observed HMBC (Figure 2) correlations from H-10 to C-7 and from H-8 to C-9 further verified this deduction. The configuration of *∆*^3^ was the same as that assigned for 1 based on the NOESY correlations (Figure 2). Moreover, H-7/H-9 were established to adopt the opposite orientation due to the NOESY correlation between H-7/H-10. 

**Figure 3:**
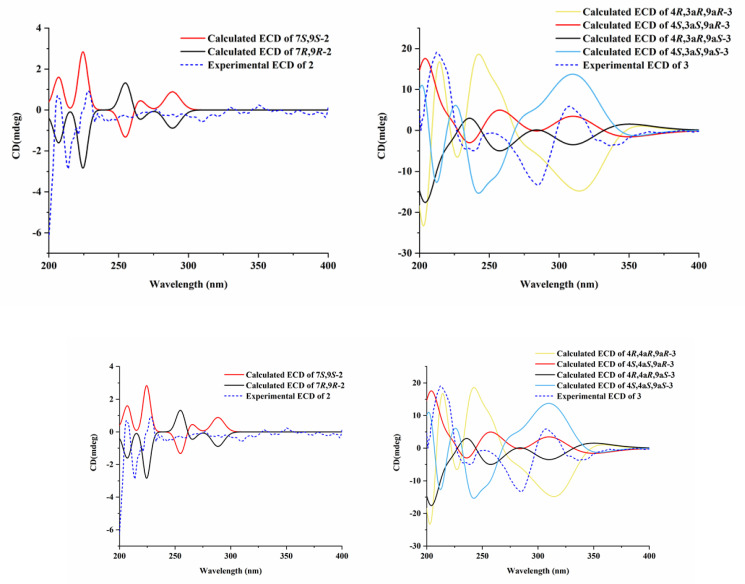
Calculated and experimental ECD spectra of **2** (**left**) and **3** (**right**).

Compound **2** possessed similar NMR data as vochysiamide B [12], with its absolute configuration undetermined. Its optical rotation was close to zero, manifesting that it was not enantiomerically pure. We tried to separate **2** and obtained an enantiomerically pure compound. Then, we attempted to assign the absolute configuration by NMR calculations performed with DP4^+^ analysis [13]. The NMR chemical shifts based on the atomic orbital (GIAO) method were predicted for four diastereomers of **2** (7R*,9R*-**2**, 7R*,9S*-**2**, 7S*,9R*-**2**, 7S*,9S*-**2**) and NMR calculations performed with DP4^+^ analysis for those four possible isomers were applied. The results indicated that 7S*,9S*-**2** was the most likely candidate structure, with a 72.77% DP4^+^ probability (Figure S25 and Table S1). If we consider the NOESY correlation between H-7/H-10, two diastereomers of **2** (7R*,9R*-**2**, 7S*,9S*-**2**) were predicted through NMR calculations. 7S*,9S*-**2** also was the most likely candidate structure, with a 99.88% DP4^+^ probability (Figure S26 and Table S2). Based on these expositional descriptions, we determined the relative configuration of **2** as 7*S*,9*S*. Consequently, ECD calculations were performed using the time-dependent density functional theory (TDDFT) methodology at the B3LYP/6-311G* level to the absolute configuration of **2**. The high similarity between the calculated ECD curve of 7*S*,9*S*-**2** and its experimental curve (Figure 3) unambiguously confirmed the absolute configuration of **2** as 7*S*,9*S*. The optical rotation value of 7*S*,9*S*-**2** is +12.38, so it is named as (+)-vochysiamide B.

Compound **3** was obtained as a brown powder, and had the molecular formula C_13_H_13_O_6_ (7 degrees of unsaturation) as determined by HRESIMS data, which showed a deprotonated ion peak at *m/z* 265.0720 ([M − H]^−^). The ^1^H NMR data (Table 1) along with the ^13^C NMR data displayed two aromatic/olefinic methines (*δ*_C/H_ 105.0/6.73, C-5; 99.2/6.41, C-7), one methyl (*δ*_C/H_ 55.8/3.84,C-10), two methylenes (*δ*_C/H_ 74.8/3.68, 3.54, C-3; 70.7/4.20, 4.12, C-1), one methine (*δ*_C/H_ 71.0/4.95, C-4), one carbonyl (*δ*_C_ 201.5, C-9), and three hydroxyls (*δ*_H_ 12.77, 6.13, 5.20). Moreover, OH-4 (*δ*_H_ 6.13) and OH-3a (*δ*_H_ 5.61) were identified by the HMBC correlations (Figure 2) from H-4 to C-3a and C-4a, from OH-4 to C-4, C-3a, and C-4a, and from OH-3a to C-3 and C-9a. The HMBC correlations from H-5 to C-7 and C-8a, as well as from H-7 to C-8a, also further verified the consistency of the structure. So, **3** was confirmed with the same plane structure with 3a,9a-deoxy-3a hydroxy-1-dehydroxyarthrinone, with its absolute configuration unconfirmed in the reference [14].

We tried to assign the absolute configuration by combining 2D NMR data and NMR calculations performed with DP4^+^ analysis. Subsequently, the relative configurations of C-4, C-3a, and C-9a in **3** were confirmed by comprehensive analysis of the NOESY spectrum (Figure 2). The NOESY cross-peaks of OH-4 and OH-3a suggested reasonable cis conformations (4R*,3aR* or 4S*,3aS*). Thus, the NMR chemical shifts based on the GIAO method were predicted for four diastereomers of **3** (4R*,3aR*,9aR*-**3**, 4R*,3aR*,9aS*-**3**, 4S*,3aS*,9aR*-**3**, 4S*,3aS*,9aS*-**3**) and NMR calculations performed with DP4^+^ analysis for those four possible isomers were applied. The results showed that 4S*, aS*,9aR*-**3** was the most likely candidate structure, with a 99.24% DP4^+^ probability (Figure S27 and Table S3). Based on these expositional descriptions, the relative configuration of **3** could be identified as 4*S*,3a*S*,9a*R*. Accordingly, the experimental ECD curves of **3** showed nice agreement with the calculated ECD curve for 4*S*,3a*S*,9a*R*-**3** (Figure 3). Thus, compound **3** was identified as 4*S*,3a*S*,9a*R*-3a,9a-deoxy-3a hydroxy-1-dehydroxyarthrinone (**3**).

The other fifteen compounds (Figure 1) were elucidated as 2, 3, 6, 8-tetrahydroxy-1-methylxanthone (**4**) [15], (+)-griseofulvin (**5**) [16], (*R*)-(-)-5-hydroxymethylmellein (**6**) [17], bungein A (**7**) [18], 3-ethylpyrazine-2,5-dipropanoic acid (**8**) [19], (*S*)-4-hydroxy-2,3-dimethyl-4-pentyl-*γ*-lactone (**9**) [20], (*R*)-2-hydroxy-3-phenylpropanoic acid (**10**) [21], 1-phenylbutane-2,3-diol (**11**) [22], *p*-hydroxybenzaldehyde (**12**) [23], 4-methoxyphenylacetic acid (**13**) [24], 4-hydroxyacetophenone (**14**) [25], 4-hydroxy phenethyl acetate (**15**) [26], methyl 2-hydroxy-3-(4’-hydroxy)-phenyl propionate (**16**) [27], protocatechoic acid (**17**) [28], and apocynin (**18**) [29] by comparing their NMR and MS data with those reported in the literature. 

**Figure 4:**
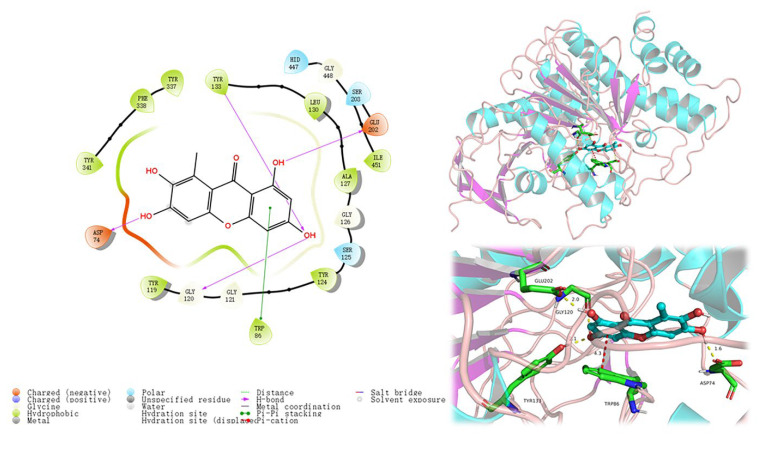
2D and 3D models of **4** with AChE (4EY7) predicted by in silico molecular docking.

All the isolated compounds were assessed for their enzyme inhibitory activities against acetylcholinesterase (AChE). Compounds **3**, **4**, **6**, **8**, **11**, and **15** exhibited obvious inhibition against AChE with an inhibitory rate more than 80% at 50 μg/mL, comparative to the positive control tacrine with an inhibitory rate 83.7% at 50 μg/mL (Table 2). Among them, 2,3,6,8-tetrahydroxy-1-methylxanthone (**4**) showed the strongest activity relatively, with an inhibitory rate 86% at 50 μg/mL. Subsequently, molecular docking analysis was conducted to investigate the binding modes between active compounds and AChE. Compounds **2**–**12**, **15**, and **17** appeared to interact with AChE protein (PDB ID: 4EY7) perfectly with the docking scores from −6.213 to −9.383 (Table 2) (the positive ligand E20 with the docking score −12.482, and tacrine with the docking score −9.965). As shown in Figure 4, phenolic hydroxy groups of **4** (the docking score −9.383) formed four hydrogen bonds with the active site residues GLU 202, TYR 133, GLY 120, and ASP 74. Additionally, the aromatic ring of **4** formed a π-π stacking interaction with TRP 86. 

All compounds were also assessed for their antibacterial activities against five pathogenic bacteria, *Staphylococcus aureus* (ATCC 29213), *Enterococcus faecalis* (ATCC 29212), *Klebsiella pneumoniae* (ATCC 13883), *Escherichia coli* (ATCC 25922), and methicillin-resistant *Staphylococcus aureus* (MRSA). Nevertheless, no compounds displayed obvious antibacterial activities.

## 3. Materials and Methods

### 3.1. General Experimental Procedures

Optical rotations were measured on a PerkinElmer MPC 500 (Waltham, MA, USA) polarimeter. UV and ECD spectra were recorded on a Chirascan circular dichroism spectrometer (Applied Photophysics, Leatherhead Surrey, UK). IR spectra were performed on an IR Affinity-1 spectrometer (Shimadzu, Kyoto, Japan). The NMR spectra were obtained on a Bruker Avance spectrometer (Bruker, Billerica, MA, USA) operating at 500 and 700 MHz for ^1^H NMR and 125 and 175 MHz for ^13^C NMR, using tetramethylsilane as an internal standard. HRESIMS spectra were collected on a Bruker miXis TOF-QII mass spectrometer (Bruker, Billerica, MA, USA). TLC and column chromatography (CC) were performed on plates precoated with silica gel GF254 (10–40 μm) and over silica gel (200–300 mesh) (Qingdao Marine Chemical Factory, Qingdao, China) and Sephadex LH-20 (Amersham Biosciences, Uppsala, Sweden), respectively. All solvents employed were of analytical grade (Tianjin Fuyu Chemical and Industry Factory, Tianjin, China). Semipreparative HPLC was carried out using an ODS column (YMC-pack ODS-A, YMC Co., Ltd., 10 × 250 mm, 5 μm, 2.5 mL/min). The artificial sea salt was a commercial product (Guangzhou Haili Aquarium Technology Company, Guangzhou, China).

### 3.2. Fungal Strain

The *Arthrinium* sp. SCSIO 41421 was isolated from a spongia sample collected from Weizhou Island, Guangxi, China, in October 2020. The strain was stored on Muller Hinton broth (MB) agar (malt extract 15 g, artificial sea salt 24 g, and agar 18 g) slants at 4 °C, and a voucher specimen was deposited in the CAS Key Laboratory of Tropical Marine Bioresources and Ecology, South China Sea Institute of Oceanology, Chinese Academy of Sciences, Guangzhou, China. It was identified as *Arthrinium* sp. by analysis of its ITS region of the rDNA as described in the Supporting Information (GenBank database accession no. OP022423). 

### 3.3. Fermentation, Extraction and Isolation

The strain was cultured on MB agar plates at 25 °C for 7 days. The seed medium (malt extract 15 g, artificial sea salt 24 g in 1.0 L of tap distilled H_2_O, pH 7.4–7.8) in 1000 mL Erlenmeyer flasks (300 mL per flask) was inoculated with strain SCSIO 41421 and incubated at 25 °C for 3 days on a rotating shaker (180 rpm). Then, seed medium was inoculated into a 1000 mL × 80 Erlenmeyer flasks containing solid rice medium (200 g of rice, 6 g artificial sea salt, and 250 mL tap distilled H_2_O in each flask). After cultivation at 25 °C for 30 days, each culture broth was extracted with an equal volume of ethyl acetate three times and broken with an ultrasonic treatment apparatus for 10 min. The organic extract was then concentrated under vacuum to afford the crude extract (62.8 g).

The ethyl acetate extract was subjected to silica gel vacuum liquid chromatography using a step gradient elution of petroleum ether (PE)-ethyl acetate (EA) (*v*:*v* 2:8, 3:7, 4:6, 5:5, 6:4, 8:2, 0:1), EA: methyl alcohol (MeOH) (*v*:*v* 100:1, 50:1, 20:1, 0:1), to yield thirteen fractions (Frs.1~13) according to TLC profiles. Fr.4 was separated by semipreparative HPLC (35% MeCN/H_2_O, 2.5 mL/min, 210 nm) to provide **12** (34.2 mg, *t*_R_ 20 min) and **14** (2.1 mg, *t*_R_ 42 min). Compounds **13** (1.7 mg, *t*_R_ 20 min) and **15** (9.0 mg, *t*_R_ 35 min) were further obtained from Fr.4 by semipreparative HPLC (30% MeCN/H_2_O, 2.5 mL/min, 210 nm; 2% MeCN/H_2_O, 2.5 mL/min, 210 nm), respectively. Fr.4 also was purified by semipreparative HPLC (21% MeCN/H_2_O, 2.5 mL/min, 210 nm) to offer **18** (12.3 mg, *t*_R_ 12 min) and **9** (4.5 mg, *t*_R_ 15 min). Meanwhile, Fr.5 was divided into six subfractions (Frs.5-1~5-6) by ODS silica gel eluting with MeOH/H_2_O (10–100%). Then, Frs.5-3 was directly separated by semipreparative HPLC (48% MeOH/H_2_O, 2.5 mL/min, 210 nm) to yield **10** (3.2 mg, *t*_R_ 22 min). Frs.5-4 was directly separated by semipreparative HPLC (19% MeOH/H_2_O, 2.5 mL/min, 210 nm) to produce **6** (6.6 mg, *t*_R_ 26 min) and **11** (10.2 mg, *t*_R_ 40 min). Compounds **17** (4.6 mg, *t*_R_ 15 min, 28% MeOH/H_2_O, 2.5 mL/min, 210 nm), **7** (23.9 mg, *t*_R_ 17 min, 30% MeOH/H_2_O, 2.5 mL/min, 210 nm), and **2** (4.3 mg, *t*_R_ 30 min, 30% MeOH/H_2_O, 2.5 mL/min, 210 nm) were obtained from Fr.7 by ODS silica gel eluting with MeOH/H_2_O (10–100%). Fr.8 was divided into eight subfractions (Frs.5-1~5-8) by ODS silica gel eluting with MeOH/H_2_O (10–100%). Then, Frs.8-2 was directly separated by semipreparative HPLC (15% MeCN/H_2_O, 2.5 mL/min, 210 nm) to yield **1** (1.6 mg, *t*_R_ 31 min) and **16** (1.7 mg, *t*_R_ 32 min). Frs.8-6 was directly separated by semipreparative HPLC (68% MeOH/H_2_O, 2.5 mL/min, 210 nm) to yield **8** (6.1 mg, *t*_R_ 14 min). Compounds **5** (22.8 mg, *t*_R_ 12 min, 55% MeCN/H_2_O, 2.5 mL/min, 210 nm), **8** (2.5 mg, *t*_R_ 18 min, 20% MeCN/H_2_O, 2.5 mL/min, 210 nm), and **3** (4.7 mg, *t*_R_ 32 min, 20% MeCN/H_2_O, 2.5 mL/min, 210 nm) were obtained from Fr.9 by ODS silica gel eluting with MeOH/H_2_O (10–100%).

(±)-vochysiamide C (**1**): colourless oil; ${\left[\alpha \right]}_{D}^{25}\;$0.0 (*c* 0.1, MeOH); UV (MeOH) *λ*_max_ (log *ε*): 223 (3.94), 278 (1.71) nm; IR (film) *ν*_max_ 2968, 2363, 1701, 1636, 1398, 1018, 1013, 698, 528 cm^−1^; ^1^H and ^13^C NMR (Table 1). HRESIMS *m/z* 214.1077 [M + H]^+^ (calcd for C_10_H_16_NO_4_, 214.1074).

(+)-vochysiamide B (**2**): brown oil; ${\left[\alpha \right]}_{D}^{25}\;$12.38 (*c* 0.1, MeOH); UV (MeOH) *λ*_max_ (log *ε*): 211 (3.01), 222 (3.12) nm; ECD (0.3 mg/mL, MeOH) *λ*_max_ (Δ *ε*): 209 (+0.12), 221 (−1.20), 228 (+0.93); IR (film) *ν*_max_ 3347, 2943, 2835, 1701, 1659, 1449, 1429, 1117, 1020, 668, 573 cm^−1^; ^1^H and ^13^C NMR (Table 1). HRESIMS *m/z* 228.1233 [M + H]^+^ (calcd for C_11_H_18_NO_4_, 228.1230).

4*S*,3a*S*,9a*R*-3a,9a-deoxy-3a hydroxy-1-dehydroxyarthrinone (**3**): brown oil; ${\left[\alpha \right]}_{D}^{25}\;$−26.40 (*c* 0.1, MeOH); UV (MeOH) *λ*_max_ (log *ε*): 215 (3.09), 252 (2.22), 286 (2.94), 320 (2.61) nm; ECD (0.3 mg/mL, MeOH) *λ*_max_ (Δ *ε*): 212 (+19.10), 240 (−4.98), 252 (−0.63), 285 (−13.39), 308 (+5.97); IR (film) *ν*_max_ 2968, 2363, 1701, 1636, 1398, 1501, 1051, 1012, 669, 525 cm^−1^; ^1^H and ^13^C NMR (Table 1). HRESIMS *m/z* 265.0720 [M – H]^−^ (calcd for C_13_H_13_O_6_, 265.0718).

### 3.4. Measurement of AChE Inhibition Activity

AChE inhibitory activities of **1**–**18** were assessed according to the spectrophotometric method with slight modification. Tacrine was used as a positive control with the IC_50_ value of 0.068 μM [30].

### 3.5. Antibacterial Activity Assay

The antibacterial activity was assessed using K-B disc agar diffusion method. Compounds **1**–**18** were tested for their antibacterial activities against five pathogenic bacteria, *Staphylococcus aureus* (ATCC 29213), E*nterococcus faecalis* (ATCC 29212), *Klebsiella pneumoniae* (ATCC 13883), *Escherichia coli* (ATCC 25922), and Methicillin-resistant *Staphylococcus aureus* (MRSA). Ampicillin and gentamicin were used as a positive control for gram-positive and gram-negative bacteria, respectively [31]. 

### 3.6. Molecular Docking

The Schrödinger 2017-1 suite (Schrödinger Inc., New York, NY, USA) was employed to perform the docking analysis. The crystal structure of AChE (PDB code: 4EY7) [32] obtained from the Protein Data Bank was used as a starting model with all of the waters and the *N*-linked glycosylated saccharides removed, and was constructed following the Protein Prepare Wizard workflow in Maestro 11-1. The prepared ligands were then flexibly docked into the receptor using the induced-fit module with the default parameters. The figures were generated using PyMol molecular graphics software (Schrödinger 2017-1, Schrödinger Inc., New York, NY, USA).

### 3.7. NMR Computational Methods

In general, conformational analyses were carried out via random searching in Spartan’14 software and Gaussian 09 software using the MMFF94 force field with an energy cutoff of 2.5 kcal/mol for compounds **2** and **3**. The generated conformers were reoptimized using DFT method at the B3LYP/6-31G (d, p) level by the Gaussian 09 program. Subsequently, NMR shielding constants in PCM chloroform or DMSO were computed using the atomic orbital (GIAO) method at the B3LYP/6-31G (d, p) level in Gaussian 09. Boltzmann weights in chloroform or DMSO were computed through Molclus [33]. Shielding constants were used to perform DP4^+^ probability analysis [13].

### 3.8. ECD Computational Methods

The Molecular Merck force field (MMFF) and density functional theory (DFT)/TDDFT calculations of **2** and **3** were performed with the Spartan’14 and Gaussian 09 software, respectively, using default grids and convergence criteria. A MMFF conformational search generated low energy conformers with a Boltzmann population of over 5%, which were subjected to geometry optimization using the DFT method at the B3LYP/6-311G* level in MeOH using the IEFPCM model. The overall theoretical calculation of the ECD was conducted in MeOH using TDDFT at the B3LYP/6-311G* level for the stable conformers of **2** and **3**. The ECD spectra of the different conformers were generated using the Multiwfn [34] with a half-bandwidth of 0.2–0.4 eV, according to the Boltzmann-calculated contribution of each conformer after the UV correction.

## 4. Conclusions

New carboxamides, (±)-vochysiamide C (**1**) and (+)-vochysiamide B (**2**), and a new polyketide, 4*S*,3a*S*,9a*R*-3a,9a-deoxy-3a hydroxy-1-dehydroxyarthrinone (**3**), were isolated and identified from the sponge-derived fungus *Arthrinium* sp. SCSIO 41421, together with other fifteen known natural products (**4**–**18**). Their planar structures and absolute configurations were elucidated by detailed spectroscopic analysis and compared with the literature data. Several compounds exhibited obvious inhibitory activity against AchE, such as 2,3,6,8-tetrahydroxy-1-methylxanthone (**4**) with an inhibitory rate 86% at 50 μg/mL. These results would expand the bioactive natural products of sponge-derived fungus.

## Figures and Tables

**Table 1:** ^1^H and ^13^C NMR spectroscopic data of **1**–**3**.

Pos	1 (in DMSO-*d*_6_) ^a^		2 (in CDCl_3_) ^b^		3 (in DMSO-*d*_6_) ^b^	
	*δ*_C_, Type	*δ*_H_ (*J* in Hz)	*δ*_C_, Type	*δ*_H_ (*J* in Hz)	*δ*_C_, Type	*δ*_H_ (*J* in Hz)
1					70.7, CH_2_	4.20 (dd, 8.0, 1.5) 4.12 (dd, 8.0, 5.8)
2	171.4, C		172.4, C			
3	124.9, CH	6.53 (d, 1.6)	125.0, CH	6.30 (d, 1.6)	74.8, CH_2_	3.68 (d, 9.7) 3.54 (d, 9.7)
3a					147.9, C	
4	154.4, C		156.2, C		71.0, CH	4.95 (s)
4a					81.0, C	
5	171.2, C		172.6, C		105.0, CH	6.73 (dd, 2.5, 1.3)
6					166.8, C	
7	56.0, CH	3.99 (tt, 8.7, 5.8)	58.7, CH	4.07 (m)	99.2, CH	6.41 (d, 2.5)
8	58.3, CH_2_	3.56 (dt, 11.3, 5.8)	62.5, CH_2_	3.97 (dd, 12.1, 6.2) 3.91 (dd, 12.1, 4.3)	164.4, C	
8a					108.9, C	
9	58.3, CH_2_	3.63 (m)	67.3, CH	4.24 (m)	201.5, C	
9a					57.3, CH	3.04 (dd, 5.8, 1.5)
10	25.1, CH	2.71 (m)	20.9, CH_3_	1.19 (d, 6.4)	55.8, CH_3_	3.84 (s)
11	20.6, CH_3_	1.15 (d, 6.8)	26.1, CH_3_	2.85 (m)		
12	20.6, CH_3_	1.15 (d, 6.8)	20.9, CH_3_	1.24 (d, 6.9)		
13			20.9, CH_3_	1.24 (d, 6.9)		
OH		4.79 (t, 5.8)				12.77 (s, OH-8)
OH						6.13 (s, OH-4)
OH						5.61 (s, OH-3a)

^a^: ^1^H in 700 MHz and ^13^C in 175 MHz; ^b^: ^1^H in 500 MHz and ^13^C in 125 MHz.

**Table 2:** Inhibition rates of AChE and molecular docking scores of active compounds with AChE.

Compounds	Inhibition Rates of AchE	Docking Score	Glide Gscore
**2**	79.38%	−7.126	−7.126
**3**	84.22%	−7.041	−7.046
**4**	86.00%	−9.383	−9.518
**5**	78.36%	−7.097	−7.097
**6**	81.52%	−6.729	−6.800
**7**	71.34%	−5.645	−5.645
**8**	81.59%	−6.880	−6.880
**9**	75.89%	−6.574	−6.577
**10**	65.78%	−6.260	−6.260
**11**	80.53%	−5.624	−5.624
**12**	68.91%	−6.492	−6.581
**15**	81.32%	−6.398	−6.398
**17**	58.37%	−6.213	−6.213
**Others**	<50%	/	/

## Data Availability

Not applicable.

## Supplementary Materials

The following supporting information can be downloaded at: https://www.mdpi.com/article/10.3390/md20080475/s1, The ITS gene sequence data of *Arthrinium* sp. SCSIO 41421. Figures S1–S24: NMR, HRESIMS, UV and CD spectra of **1**–**3**; Figures S25–S27: Linear correlation plots of calculated-experimental ^13^C NMR chemical shift values with DP4^+^ analyses for potential configurations of **2** and **3**; Tables S1–S3: DP4^+^ analysis of experimental and calculated NMR chemical shifts of the isomers of **2** and **3**. The physicochemical data of compounds **1**–**18**.

## References

[1] Zhang B., Zhang T., Xu J., Lu J., Qiu P., Wang T., Ding L. (2020). Marine sponge-associated fungi as potential novel bioactive natural product sources for drug discovery. Mini Rev. Med. Chem..

[2] Jansen N., Ohlendorf B., Erhard A., Bruhn T., Bringmann G., Imhoff J.F., Helicusin E. (2013). isochromophilone X and isochromophilone XI: New chloroazaphilones produced by the fungus *Bartalinia robillardoides* strain LF550. Mar. Drugs.

[3] Kong F., Zhao C., Hao J., Wang C., Wang W., Huang X., Zhu W. (2015). New α-glucosidase inhibitors from a marine sponge-derived fungus, *Aspergillus* sp. OUCMDZ-1583. RSC Adv..

[4] Zhao Y., Liu D., Proksch P., Yu S., Lin W. (2016). Isocoumarin derivatives from the sponge-associated fungus *Peyronellaea glomerata* with antioxidant activities. Chem. Biodivers..

[5] Kuppers L., Ebrahim W., El-Neketi M., Ozkaya F.C., Mandi A., Kurtan T., Orfali R.S., Muller W.E.G., Hartmann R., Lin W. (2017). Lactones from the sponge-derived fungus *Talaromyces rugulosus*. Mar. Drugs.

[6] Guo T.T., Song M.M., Han W.R., Zhu J.H., Liu Q.C., Wang J.F. (2021). New *N*-methyl-4-quinolone alkaloid and citrinin dimer derivatives from the sponge-derived fungus *Penicillium* sp. SCSIO 41303. Phytochem. Lett..

[7] Liu Y., Ding L., He J., Zhang Z., Deng Y., He S., Yan X. (2021). A new antibacterial chromone from a marine sponge-associated fungus *Aspergillus* sp. LS57. Fitoterapia.

[8] Elissawy M., Ebada S.S., Ashour M.L., Özkaya F.C., Ebrahim W., Singab A.B., Proksch P. (2017). Spiroarthrinols A and B, two novel meroterpenoids isolated from the sponge-derived fungus *Arthrinium* sp.. Phytochem. Lett..

[9] Shu Y., Wang J.P., Li B.X., Gan J.L., Ding H., Liu R., Cai L., Ding Z.T. (2022). Bioactive cytochalasans from the fungus *Arthrinium arundinis* DJ-13. Phytochemistry.

[10] Sharma R., Kulkarni G., Sonawane M.S., Shouche Y.S. (2014). A new endophytic species of *Arthrinium* (Apiosporaceae) from *Jatropha podagrica*. Mycoscience.

[11] Ebada S.S., Schulz B., Wray V., Totzke F., Kubbutat M.H., Muller W.E., Hamacher A., Kassack M.U., Lin W., Proksch P. (2011). Arthrinins A-D: Novel diterpenoids and further constituents from the sponge derived fungus *Arthrinium* sp.. Bioorg. Med. Chem..

[12] Noriler S.A., Savi D.C., Ponomareva L.V., Rodrigues R., Rohr J., Thorson J.S., Glienke C., Shaaban K.A. (2019). Vochysiamides A and B: Two new bioactive carboxamides produced by the new species *Diaporthe vochysiae*. Fitoterapia.

[13] Grimblat N., Zanardi M.M., Sarotti A.M. (2015). Beyond DP4: An improved probability for the stereochemical assignment of isomeric compounds using quantum chemical calculations of NMR shifts. J. Org. Chem..

[14] Whyte A.C., Gloer K.B., Gloer J.B., Korster B., Malloch D. (1997). New antifungal metabolites from the coprophilous fungus *Cercophora sordarioides*. Can. J. Chem..

[15] Wang C.F., Guan F.F., Du S.Y., Wei M.Y., Wang C.Y., Shao C.L. (2016). Two polyhydroxy xanthones and their antiviral activity from gorgonian coral-derived fungus *Arthrinium* sp.. Chin. J. Mar. Drugs.

[16] Liu S.Z., Zhao W.M. (2010). Chemical constituents of medicinal fungus *Shiraia bambusicola*. Chin. Tradit. Herbal Drugs.

[17] Kongprapan T., Xu X., Rukachaisirikul V., Phongpaichit S., Sakayaroj J., Chen J., Shen X. (2017). Cytosporone derivatives from the endophytic fungus *Phomopsis* sp. PSU-H188. Phytochem. Lett..

[18] Yang H., Wang J., Mei S.X., Sun H.D. (2000). A new peroxide compound from *Clerodendrum bungee*. Acta Bot. Yunnanica.

[19] Suzuki T., Yasuhara N., Ueda T. (2015). Reaction of acetaldehyde with 5-aminolevulinic acid via dihydropyrazine derivative. Chem. Pharm. Bul..

[20] Shi H., Yu S., Liu D., Ofwegen L.V., Proksch P., Lin W. (2012). Sinularones A-I, new cyclopentenone and butenolide derivatives from a marine soft coral *Sinularia* sp. and their antifouling activity. Mar. Drugs.

[21] Wang Y.Y., Luo D.Q., Shuai B.Z., Yang X.L. (2011). Secondary metabolites of endophytic fungus *Pestalotiopsis Zonata* from guava leaves. Chin. Tradit. Patent Med..

[22] Chen X.Y., Zhong W.M., Zeng Q., Wang F.Z. (2020). A preliminary study on the chemical diversity of the deep-sea derived fungus *Acrostalagmus luteoalbus* SCSIO F457 based on OSMAC strategy. Chin. J. Mar. Drugs.

[23] Li H.X., Deng T.Z., Chen Y., Feng H.J., Yang G.Z. (2007). Isolation and identification of phenolic constituents from *Juncus effusus*. Acta Pharm. Aceutica Sin..

[24] Li X.J., Gao J.M., Chen H., Zhang A.L., Tang M. (2012). Toxins from a symbiotic fungus, *Leptographium qinlingensis* associated with *Dendroctonus armandi* and their in vitro toxicities to *Pinus armandi* seedlings. Eur. J. Plant. Pathol..

[25] Li J., Kadota S., Kawata Y., Hattori M., Xu G.J., Namba T. (1992). Constituents of the roots of *Cynanchum bungei* DECNE. Isolation and structures of four new glucosides, Bungeiside-A, -B, -C, and -D. Chem. Pharm. Bull..

[26] Guan Y.Q., Cao M.M., Liu Y., Song X.M., Luo D.Q. (2014). Effect of secondary metabolites of endophytic fungus *Pestalotiopsis adusta* on HeLa cells. Chin. Tradit. Patent Med..

[27] Firdous S., Khan K., Zikr-Ur-Rehman S., Ail Z., Soomro S., Ahmad V.U., Rasheed M., Mesaik M.A., Faizi S. (2014). Isolation of phytochemicals from *Cordia rothii* (Boraginaceae) and evaluation of their immunomodulatory properties. Nat. Prod. Rep..

[28] Nechepurenko I.V., Polovinka M.P., Komarova N.I., Korchagina D.V., Salakhutdinov N.F., Nechepurenko S.B. (2008). Low molecular weight phenolic compounds from *Hedysarum theinum* roots. Chem. Nat. Compd..

[29] Li Y.J., He X., Liu Z.B., Huang Y., Lan Y.Y., Wang A.M., Wang Y.L. (2010). Chemical constituents of flowers of *Polygonum orientale*. Lishizhen Med. Mater. Med. Res..

[30] Dai Y., Li K.L., She J.L., Zeng Y.B., Wang H., Liao S.R., Lin X.P., Yang B., Wang J.F., Tao H.M. (2020). Lipopeptide epimers and a phthalide glycerol ether with AChE inhibitory activities from the marine-derived fungus *Cochliobolus Lunatus* SCSIO41401. Mar. Drugs.

[31] Cai J., Chen C.M., Tan Y.H., Chen W.H., Luo X.W., Luo L.X., Yang B., Liu Y.H., Zhou X.F. (2021). Bioactive polyketide and diketopiperazine derivatives from the mangrove-sediment-derived fungus *Aspergillus* sp. SCSIO41407. Molecules.

[32] Cheung J., Rudolph M.J., Burshteyn F., Cassidy M.S., Gary E.N., Love J., Franklin M.C., Height J.J. (2012). Structures of human acetylcholinesterase in complex with pharmacologically important ligands. J. Med. Chem..

[33] Lu T. Molclus Program, Version 1.9.9.9. http://www.keinsci.com/research/molclus.html.

[34] Lu T., Chen F.W. (2012). Multiwfn: A Multifunctional Wavefunction Analyzer. J. Comput. Chem..

