# Supplementary material for: New Carboxamides and a New Polyketide from the Sponge-Derived Fungus Arthrinium sp. SCSIO 41421
Source: Mar Drugs. 2022 Jul 25;20(8):475. doi: 10.3390/md20080475 (PMC9394276; doi:10.3390/md20080475)
Supplement: Supplementary file 1 [file marinedrugs-20-00475-s001.zip › marinedrugs-1842110-supplementary.pdf]

SUPPLEMENTARY MATERIAL

New carboxamides and a new polyketide from the sponge-derived fungus *Arthrinium* sp. SCSIO 41421

Jianglian She [1,2,3], Yi Chen [1,2], Yuxiu Ye [4], Xiuping Lin [1], Bin Yang [1], Jiao Xiao [5], Yonghong Liu [1,2,3], Xuefeng Zhou [1,2,3*]

[1] CAS Key Laboratory of Tropical Marine Bio-resources and Ecology, Guangdong Key Laboratory of Marine Materia Medica, South China Sea Institute of Oceanology, Chinese Academy of Sciences, Guangzhou 510301, China; shejianglian20@mails.ucas.ac.cn (J. S.); 13620281931@163.com (Y. C.); xiupinglin@scsio.ac.cn (X. L.); yangbin@scsio.ac.cn (B. Y.); yonghongliu@scsio.ac.cn (Y. L.)

[2] University of Chinese Academy of Sciences, Beijing 100049, China

[3] Southern Marine Science and Engineering Guangdong Laboratory (Guangzhou), Guangzhou 511458, China

[4] Institute of Marine Drugs, Guangxi University of Chinese Medicine, Nanning 530200, China; 18877548173@163.com (Y. Y.)

[5] Wuya College of Innovation, Shenyang Pharmaceutical University, Shenyang 110016, China; xj110121@126.com (J. X.)

* Correspondence: xfzhou@scsio.ac.cn (X.Z.)

Contents

The ITS gene sequence data of *Arthrinium* sp. SCSIO 41421.
Figure S1. The ^{1}H NMR spectrum of compound 1 in DMSO-d_6.
Figure S2. The ^{13}C NMR spectrum of compound 1 in DMSO-d_6.
Figure S3. The HSQC spectrum of compound 1 in DMSO-d_6.
Figure S4. The HMBC spectrum of compound 1 in DMSO-d_6.
Figure S5. The ^{1}H-^{1}H COSY spectrum of compound 1 in DMSO-d_6.
Figure S6. The NOESY spectrum of compound 1 in DMSO-d_6.
Figure S7. The HRESIMS spectrum of compound 1 in CH_3OH.
Figure S8. The UV and CD spectrums of compound 1 in CH_3OH.
Figure S9. The ^{1}H NMR spectrum of compound 2 in $CDCl_3$.
Figure S10. The ^{13}C NMR spectrum of compound 2 in $CDCl_3$.
Figure S11. The HSQC spectrum of compound 2 in $CDCl_3$.
Figure S12. The HMBC spectrum of compound 2 in $CDCl_3$.
Figure S13. The ^{1}H-^{1}H COSY spectrum of compound 2 in $CDCl_3$.
Figure S14. The NOESY spectrum of compound 2 in $CDCl_3$.
Figure S15. The HRESIMS spectrum of compound 2 in CH_3OH.
Figure S16. The UV spectrum of compound 2 in CH_3OH.
Figure S17. The ^{1}H NMR spectrum of compound 3 in DMSO-d_6.
Figure S18. The ^{13}C NMR spectrum of compound 3 in DMSO-d_6.
Figure S19. The HSQC spectrum of compound 3 in DMSO-d_6.
Figure S20. The HMBC spectrum of compound 3 in DMSO-d_6.
Figure S21. The ^{1}H-^{1}H COSY spectrum of compound 3 in DMSO-d_6.
Figure S22. The NOESY spectrum of compound 3 in DMSO-d_6.
Figure S23. The HRESIMS spectrum of compound 3 in CH_3OH.
Figure S24. The UV spectrum of compound 3 in CH_3OH.
Figure S25. Linear correlation plots of calculated-experimental ^{13}C NMR chemical shift values with DP4$^+$ analyses for potential configurations of compound 2 (Isomer 1: (7*R, 9*R**)-2, Isomer 2: (7*R**, 9*S**)-2, Isomer 3: (7*S**, 9*R**)-2 and Isomer 4: (7*S**, 9*S**)-2).**
Table S1. DP4$^+$ analysis of experimental and calculated NMR chemical shifts of Isomer 1: (7*R, 9*R**)-2, Isomer 2: (7*R**, 9*S**)-2, Isomer 3: (7*S**, 9*R**)-2 and Isomer 4: (7*S**, 9*S**)-2.**
Figure S26. Linear correlation plots of calculated-experimental ^{13}C NMR chemical shift values with DP4$^+$ analyses for potential configurations of compound 2 (Isomer 1: (7*R, 9*R**)-2 and Isomer 2: (7*S**, 9*S**)-2).**
Table S2. DP4$^+$ analysis of experimental and calculated NMR chemical shifts of Isomer 1: (7*R, 9*R**)-2 and Isomer 2: (7*S**, 9*S**)-2.**
Figure S27. Linear correlation plots of calculated-experimental ^{13}C NMR chemical shift values with DP4$^+$ analyses for potential configurations of compound 3.
Table S3. DP4$^+$ analysis of experimental and calculated NMR chemical shifts of Isomer 1: (4*R, 4a*R**, 9a*R**)-3, Isomer 2: (4*S**, 4a*S**, 9a*R**)-3, Isomer 3: (4*R**, 4a*R**, 9a*S**)-3, and Isomer 4: (4*S**, 4a*S**, 9a*S**)-3.**
The physicochemical data of compounds 1–18.

The ITS gene sequence data of *Arthrinium* sp. SCSIO 41421.

GGGTATTCCTACCTGATCCGAGGTCAACCACTAAAAATTGGGGGTTTTATGGCGGG AGGACAGAGCCTTACAGAAGCGAGAAATAAATTTACTACGCTCAGAGGACAACTA TCGCTCCGCCACTGTCTTTAAGGAACTACAGTACAGTAGATTCCCAACACTAAGCT AGGCTTAAGGGTTGAAATGACGCTCGAACAGGCATGCCCACCAGAATACTGATGG GCGCAATGTGCGTTCAAAGATTCGATGATTCACTGAATTCTGCAATTCACATTACTT ATCGCATTTCGCTGCGTTCTTCATCGATGCCAGAACCAAGAGATCCGTTGTTGAAA GTTTTAATTATTAAAATAATACGCTCAGAAGATACAATAAAACAAGAGTTTAGTGTC CACCGGCGGGCTGCGCGGGAGTGGTGCAGGGTAAGCTACAGGGTAGCCTACAGG GTAGCTACCGGGTAACCTACAGGGTAGGCTACAGGGTAGCACCCGACCTGCCTCC GTACGCAGTTCACGCCGAGGCATAACTGGTAAGTTAACAAATGGTGTGG

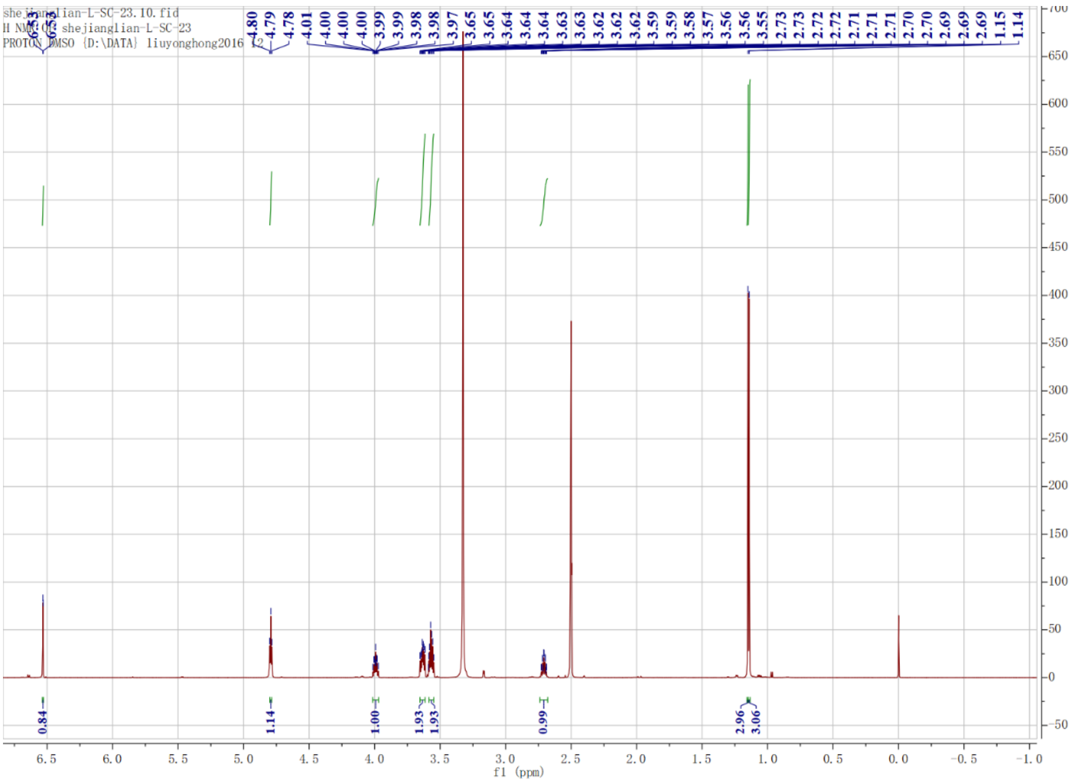

Figure S1. The ^{1}H NMR spectrum of compound 1 in DMSO-d_6.

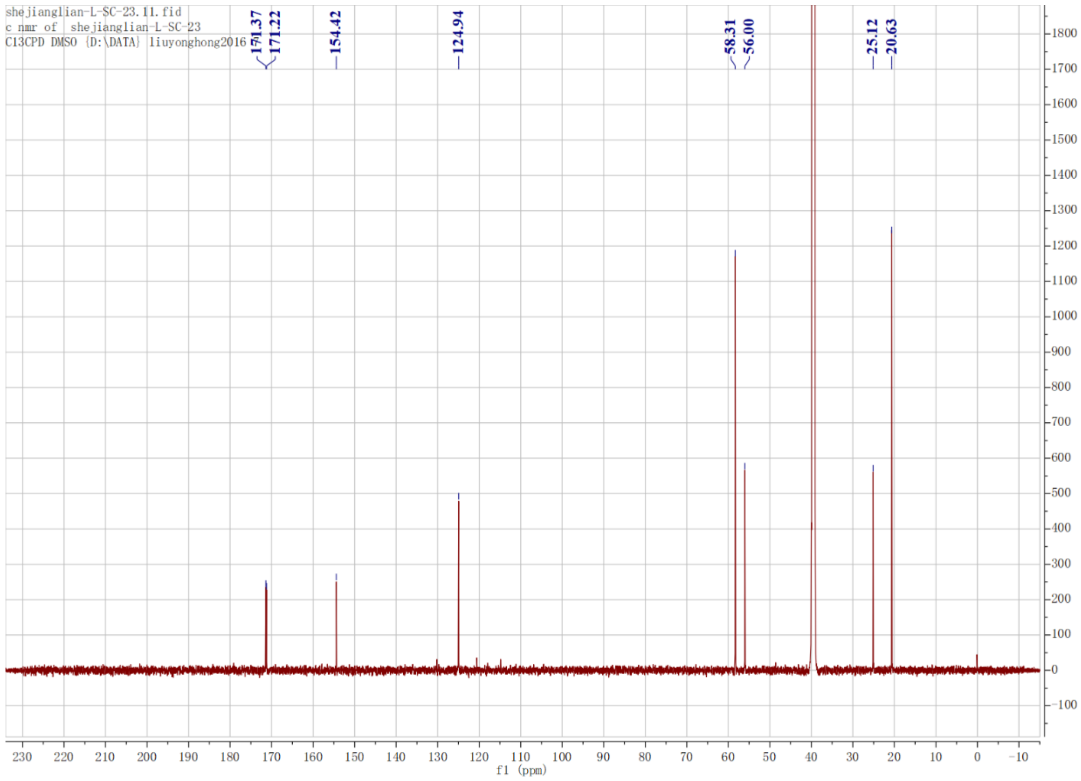

Figure S2. The ^{13}C NMR spectrum of compound 1 in DMSO-d_6.

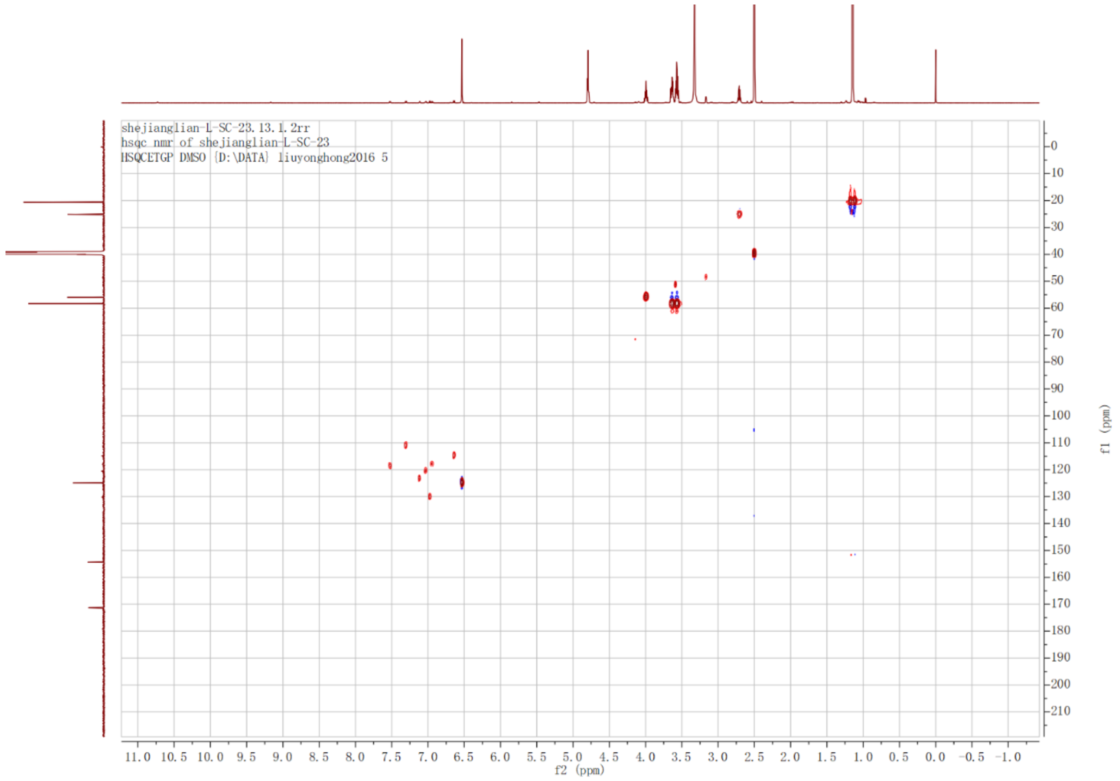

Figure S3. The HSQC spectrum of compound 1 in DMSO-d_6.

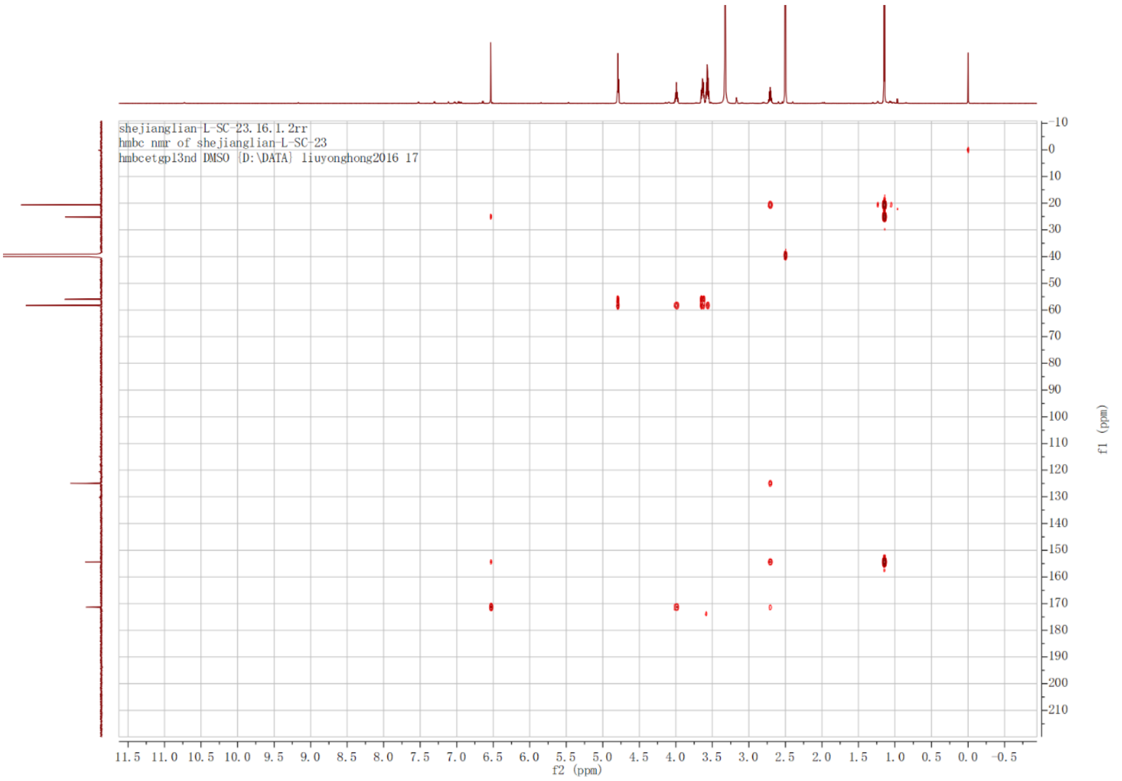

Figure S4. The HMBC spectrum of compound 1 in DMSO-*d*6.

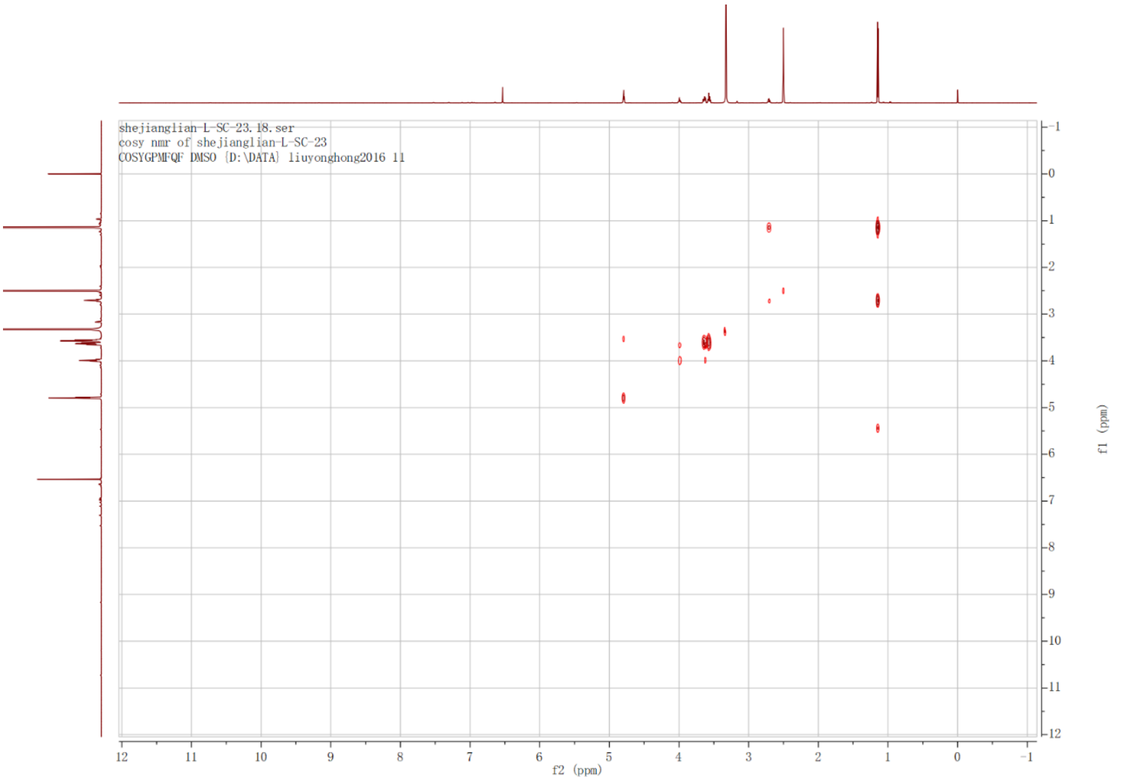

Figure S5. The ^{1}H-^{1}H COSY spectrum of compound 1 in DMSO-*d*6.

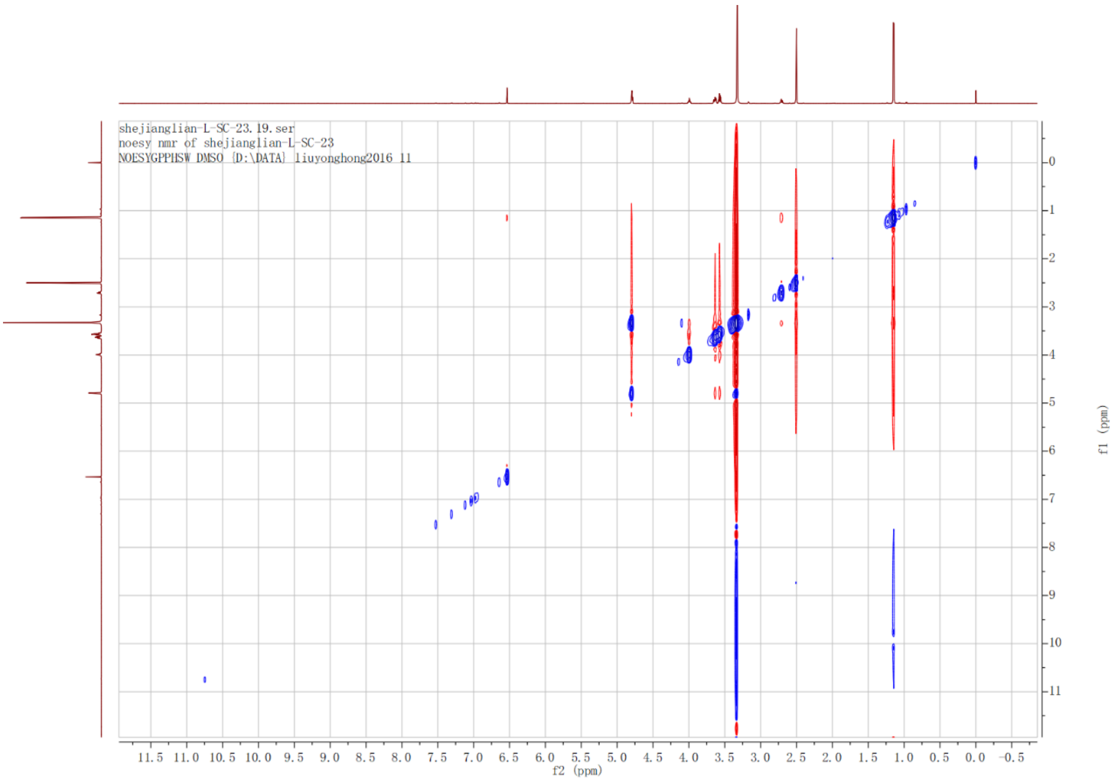

Figure S6. The NOESY spectrum of compound 1 in DMSO-*d*$_6$.

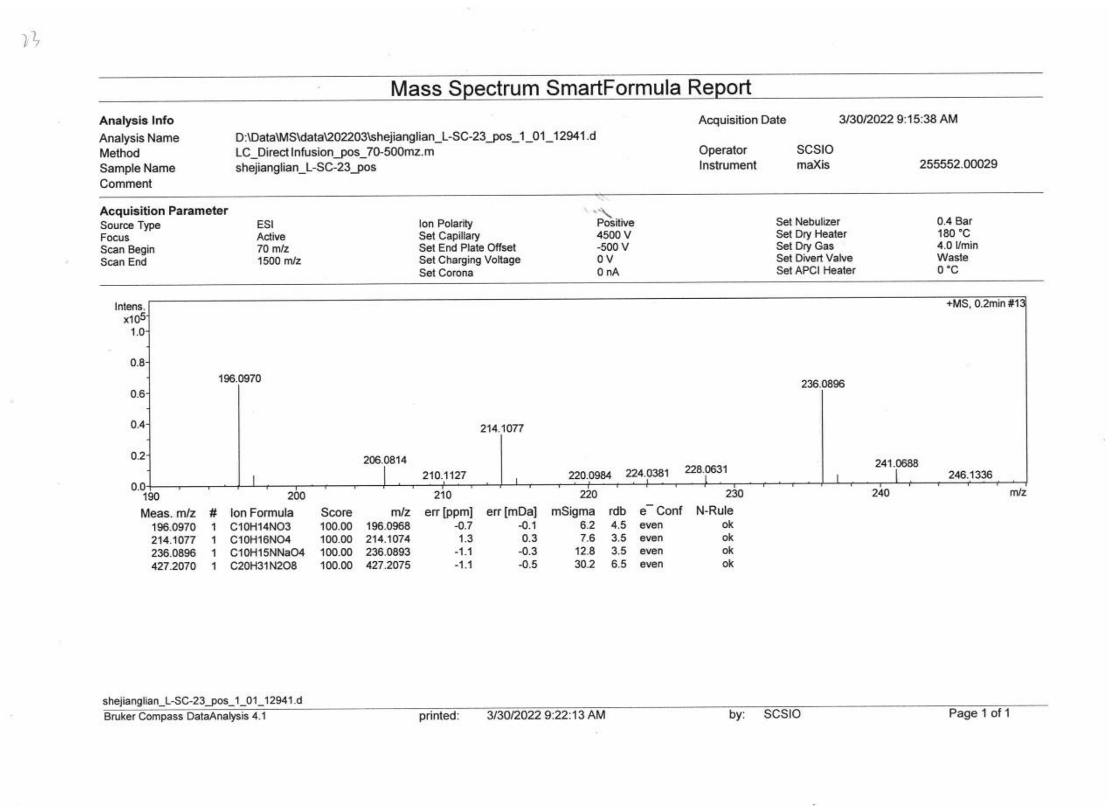

Figure S7. The HRESIMS spectrum of compound 1 in CH_3OH.

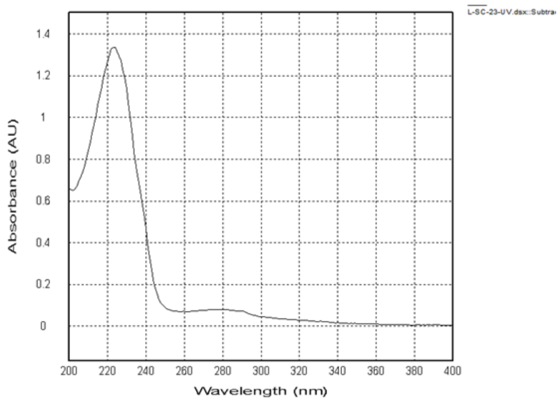

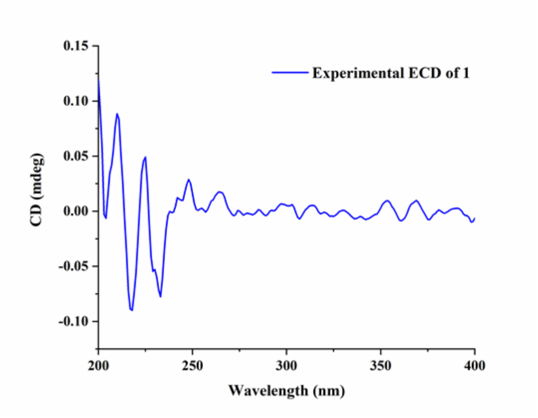

Figure S8. The UV and CD spectrums of compound 1 in CH_3OH.

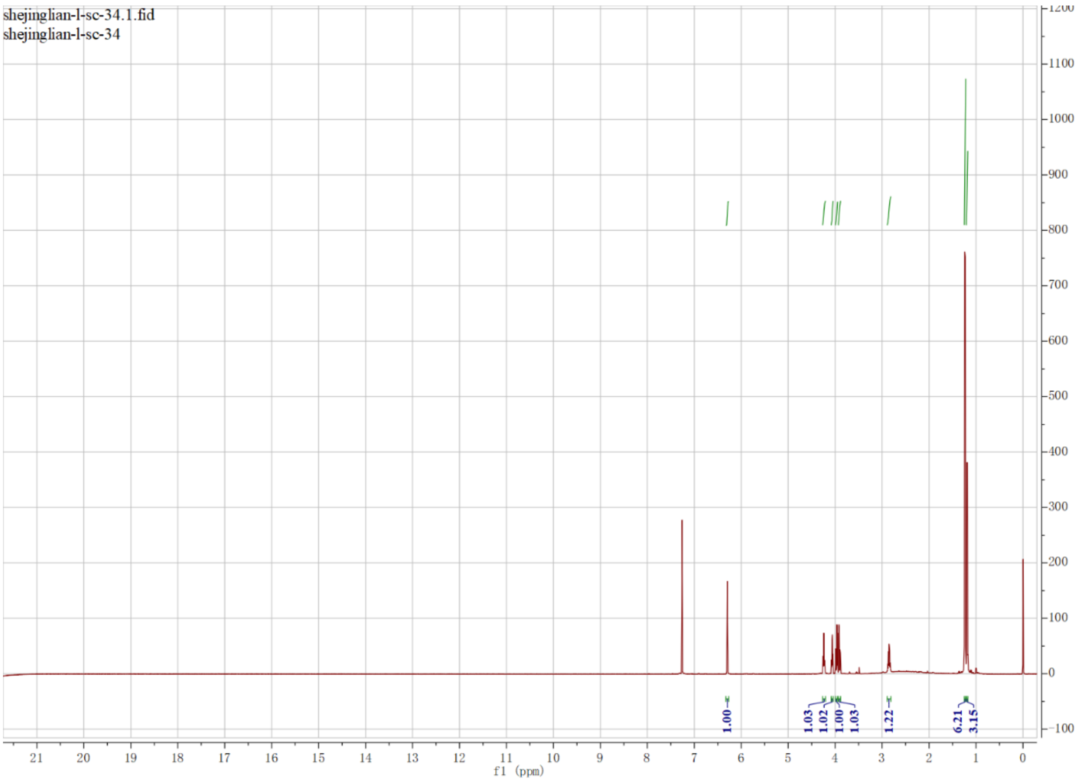

Figure S9. The ^1H NMR spectrum of compound 2 in $CDCl_3$.

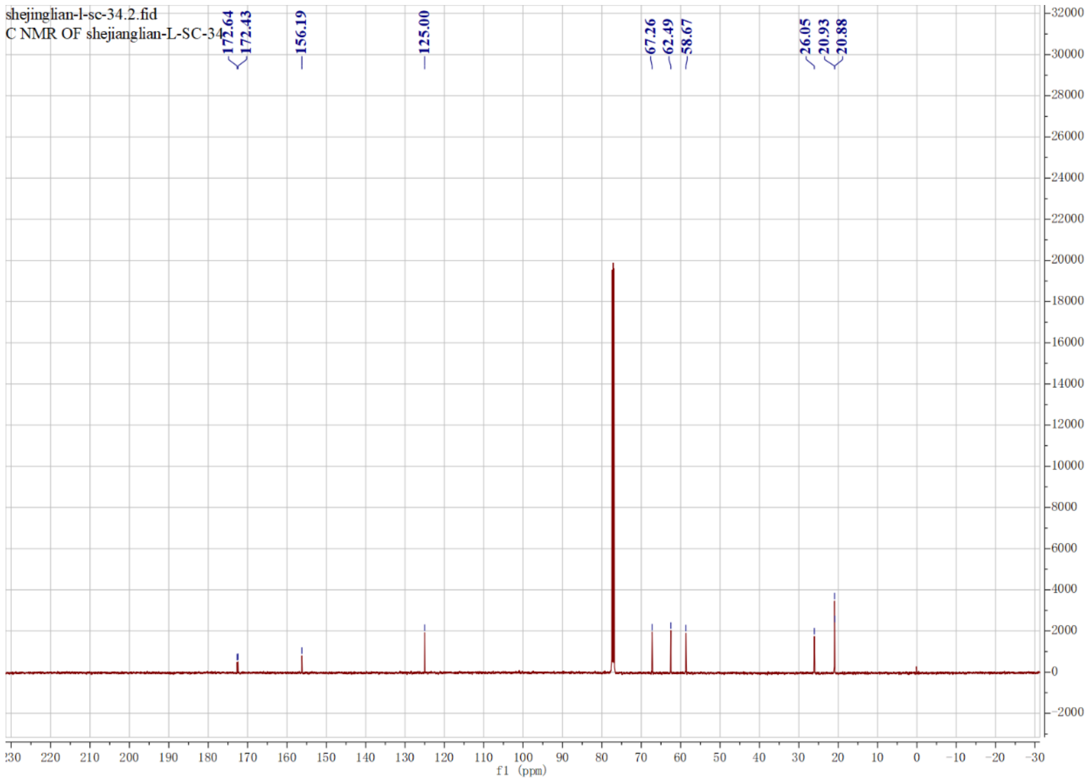

Figure S10. The ^{13}C NMR spectrum of compound 2 in $CDCl_3$.

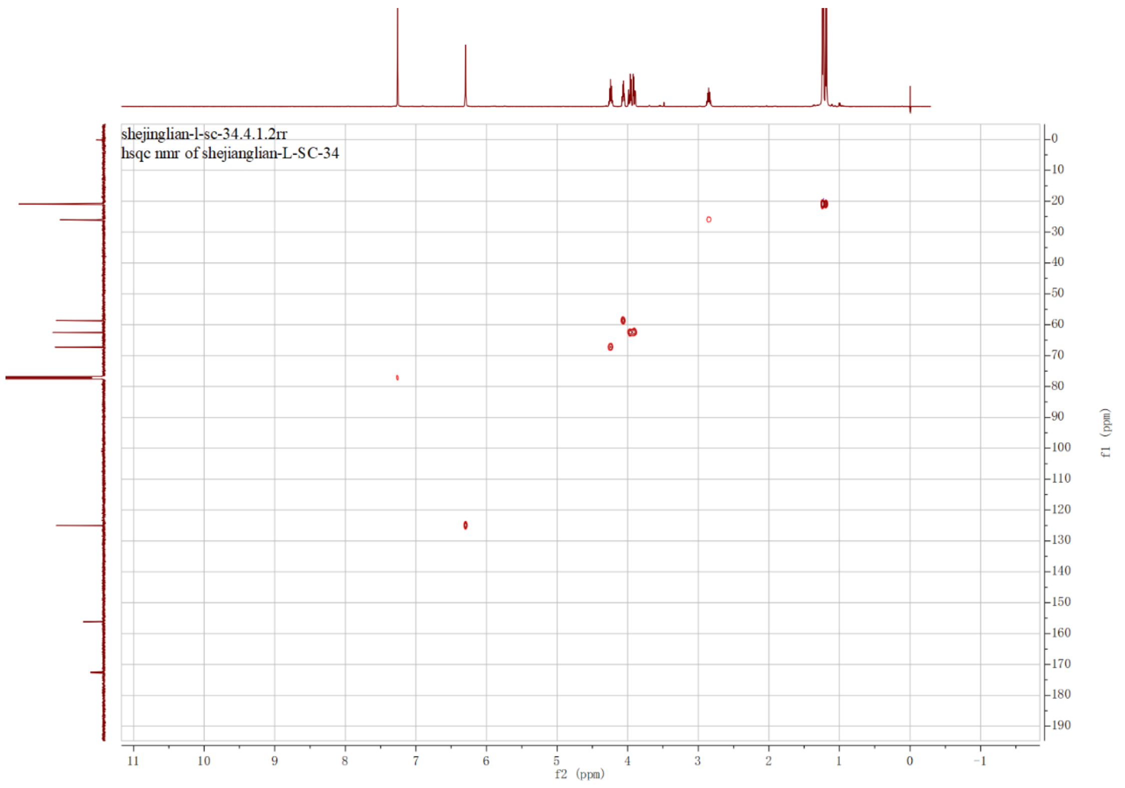

Figure S11. The HSQC spectrum of compound 2 in $CDCl_3$.

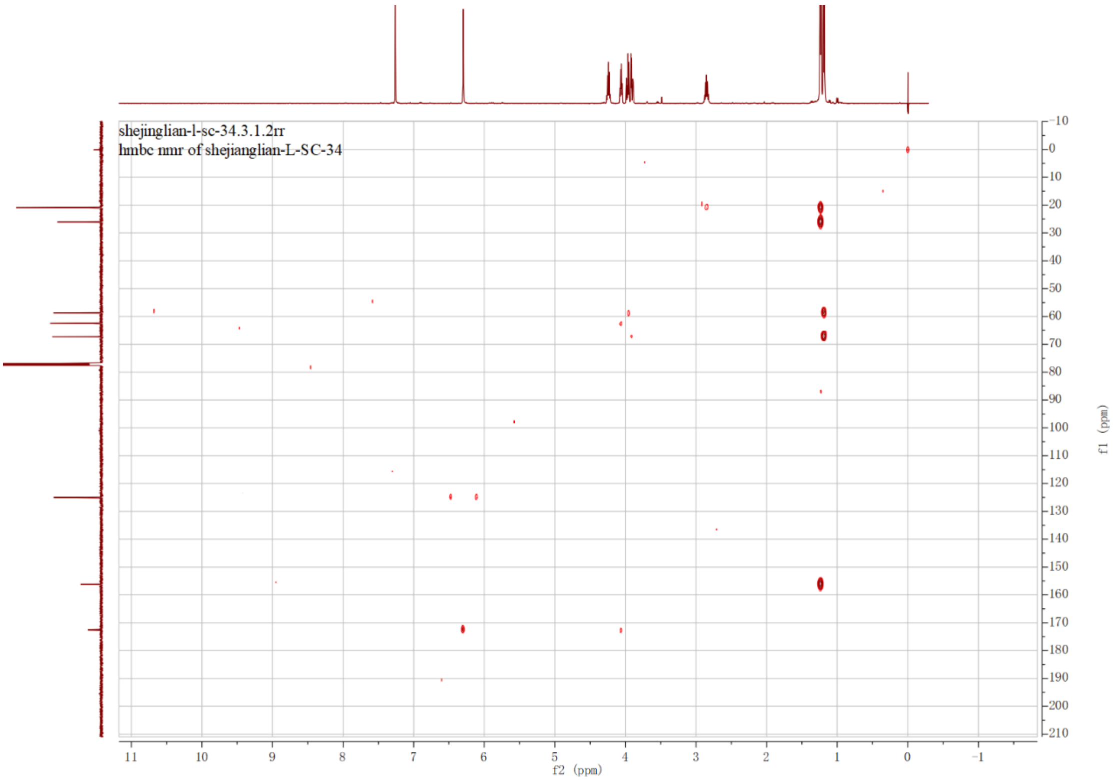

Figure S12. The HMBC spectrum of compound 2 in $CDCl_3$.

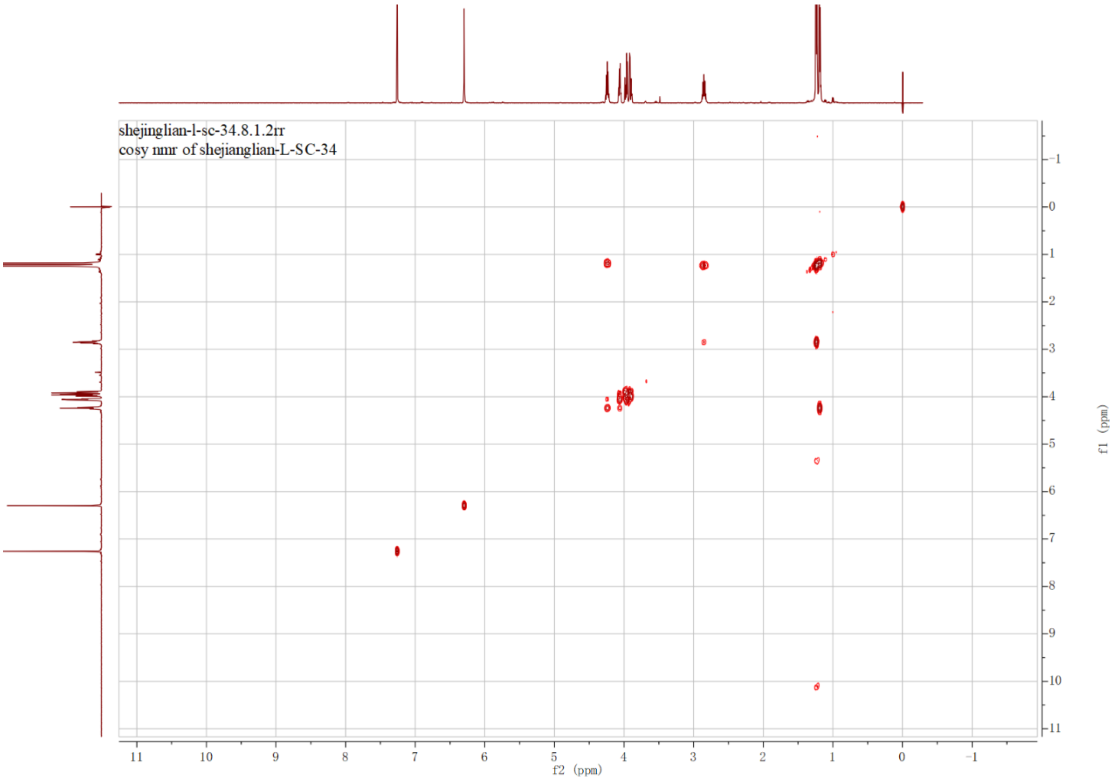

Figure S13. The ^1H-^1H COSY spectrum of compound 2 in $CDCl_3$.

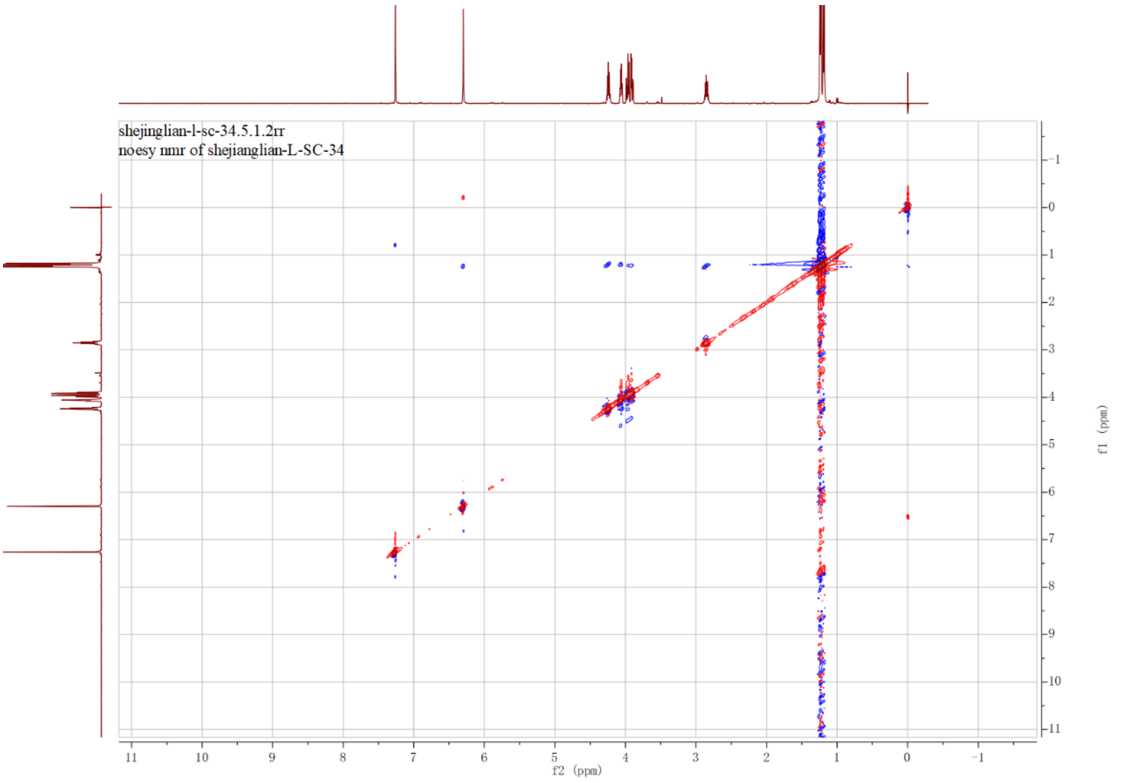

Figure S14. The NOESY spectrum of compound 2 in $CDCl_3$.

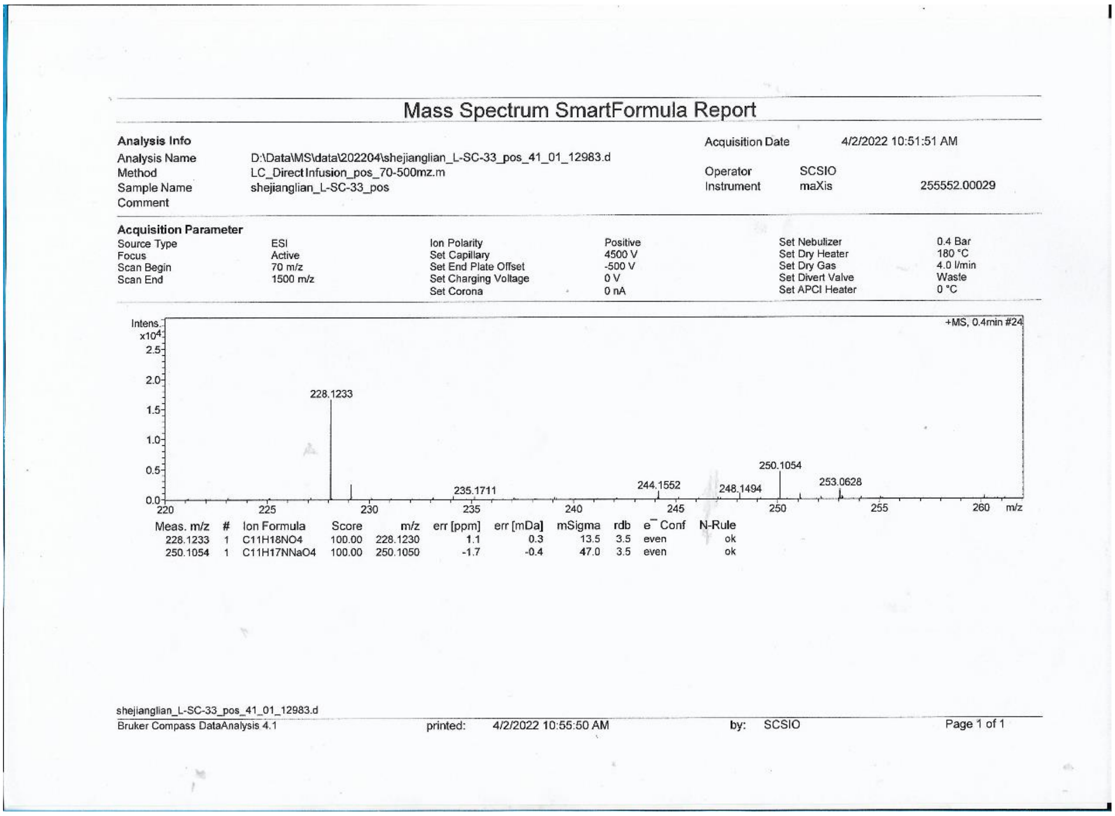

Figure S15. The HRESIMS spectrum of compound 2 in CH_3OH.

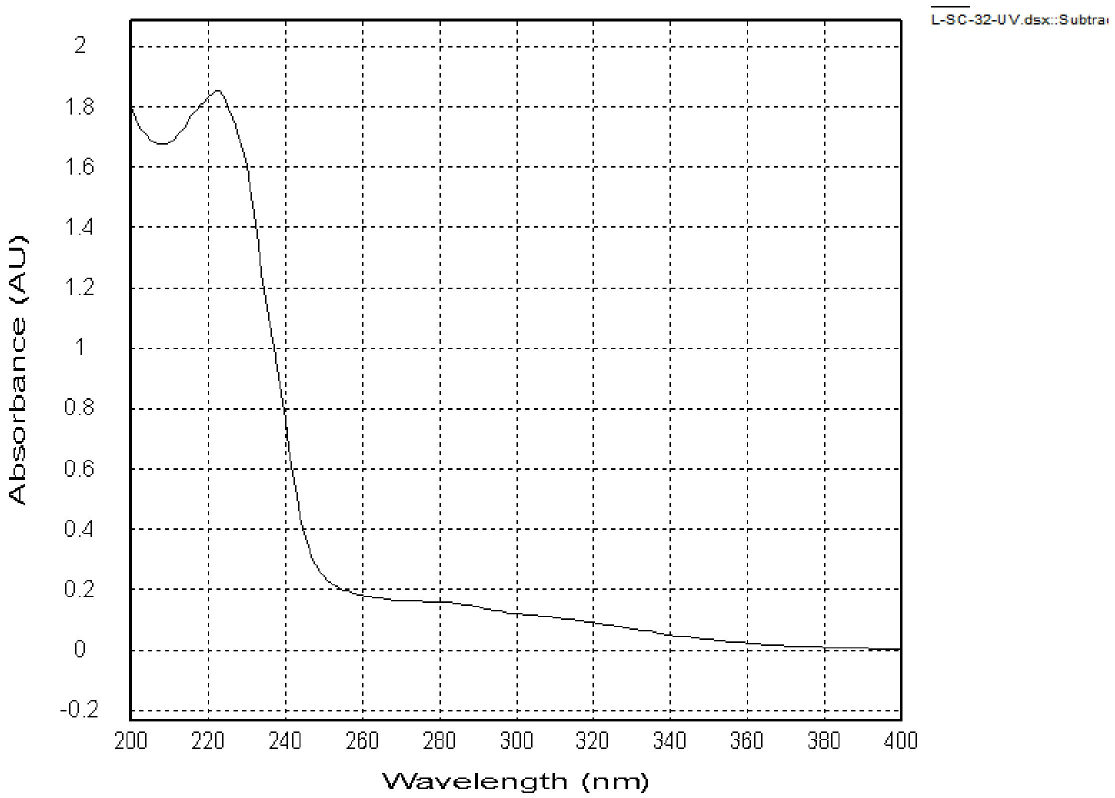

Figure S16. The UV spectrum of compound 2 in CH_3OH.

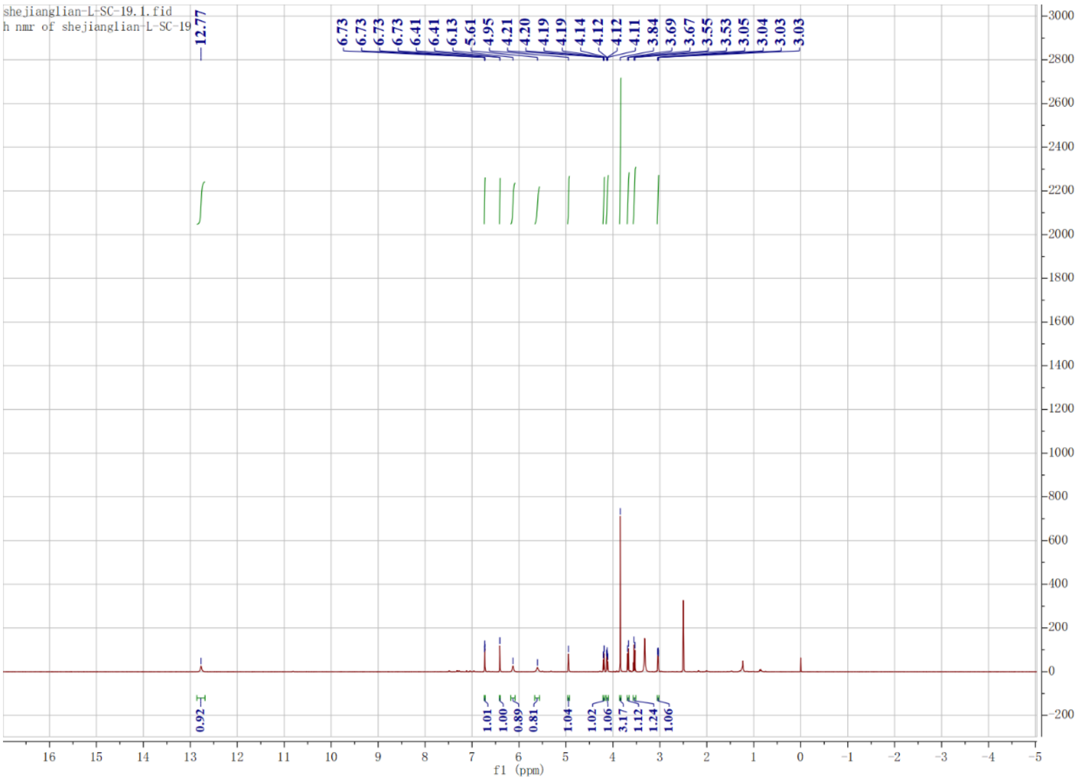

Figure S17. The ^{1}H NMR spectrum of compound 3 in DMSO-*d*$_6$.

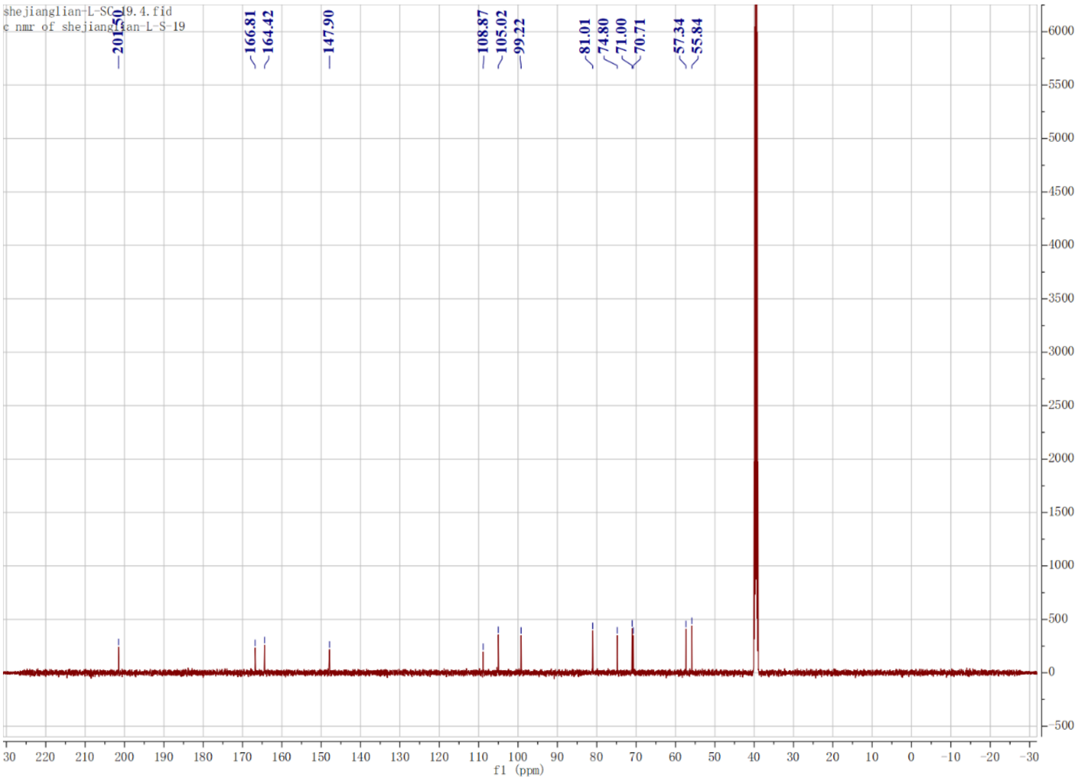

Figure S18. The ^{13}C NMR spectrum of compound 3 in DMSO-d_6.

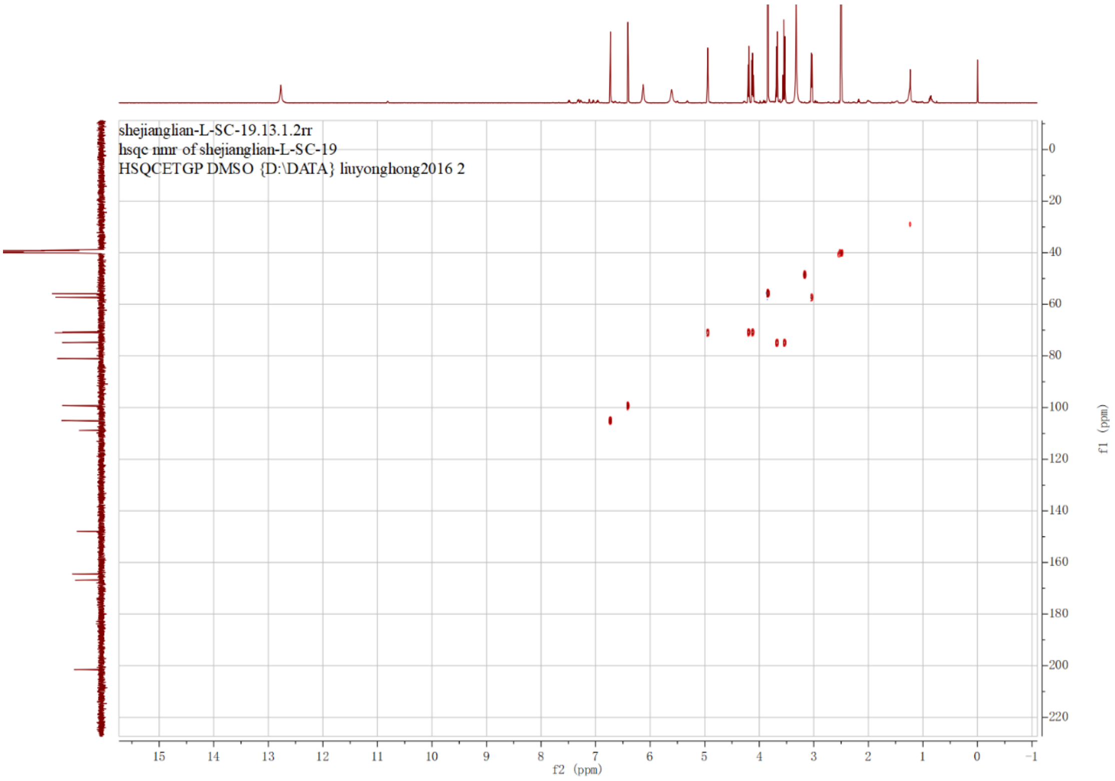

Figure S19. The HSQC spectrum of compound 3 in DMSO-d_6.

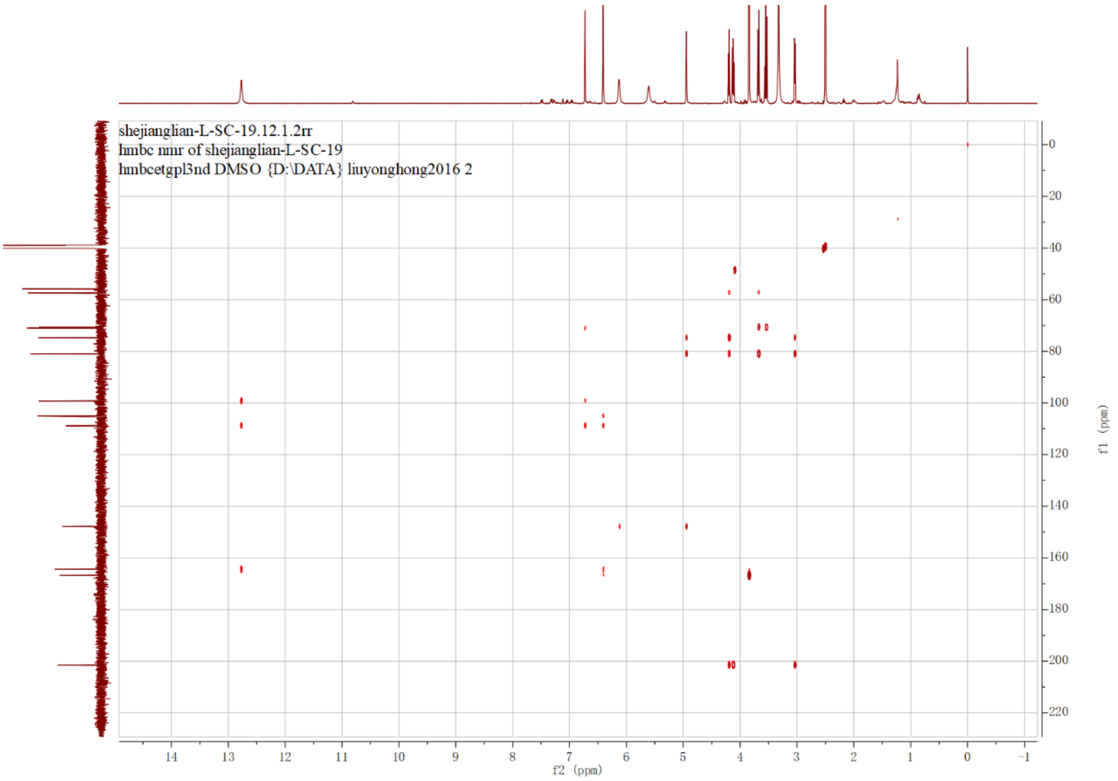

Figure S20. The HMBC spectrum of compound 3 in DMSO-d_6.

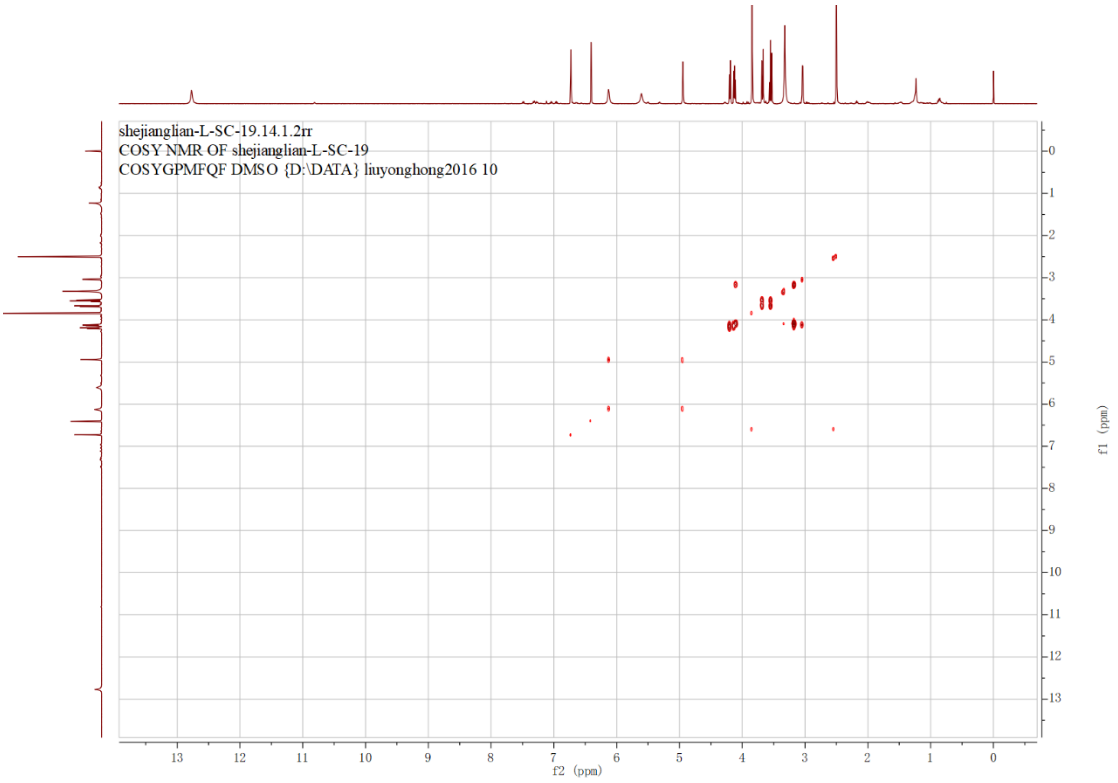

Figure S21. The ^{1}H-^{1}H COSY spectrum of compound 3 in DMSO-d_6.

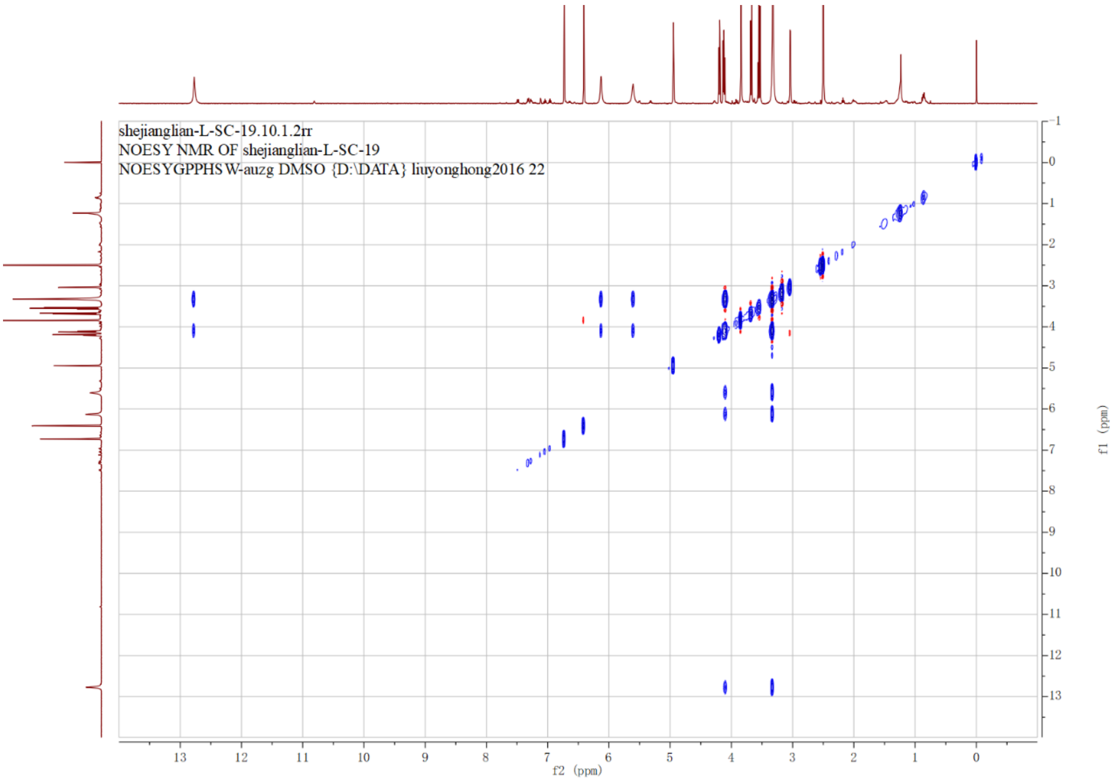

Figure S22. The NOESY spectrum of compound 3 in DMSO-*d*$_6$.

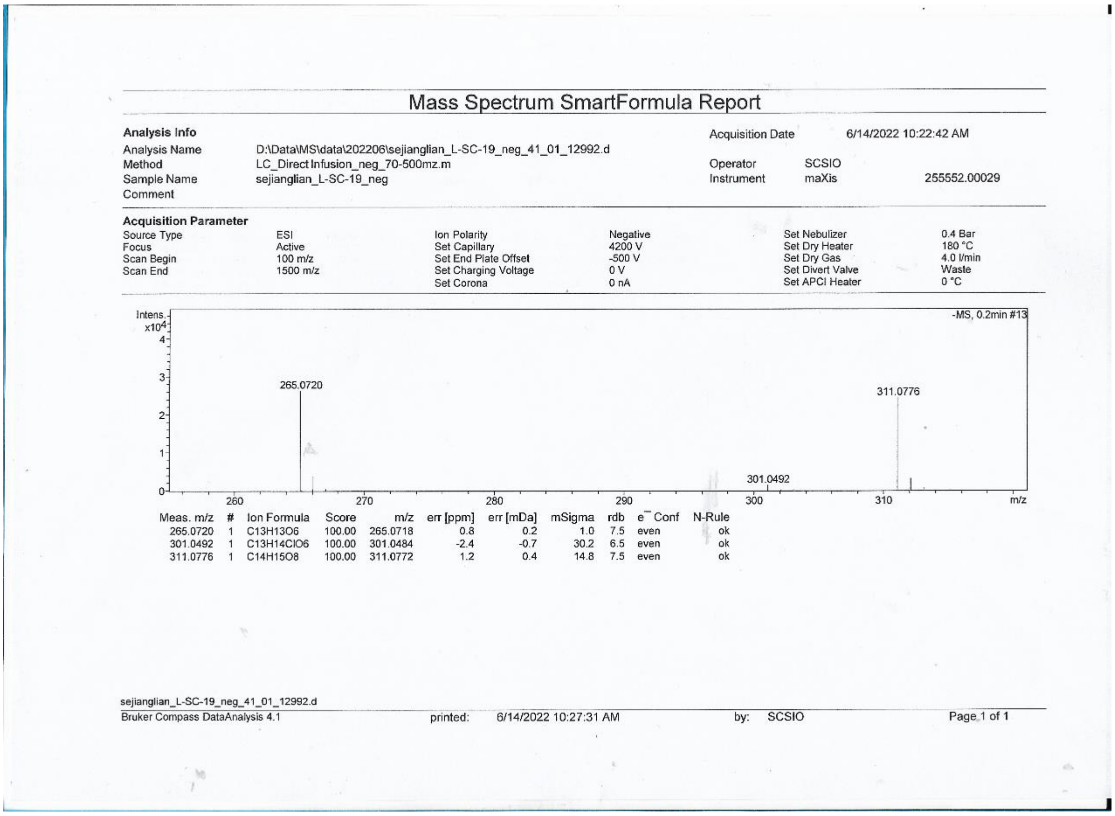

Figure S23. The HRESIMS spectrum of compound 3 in CH_3OH.

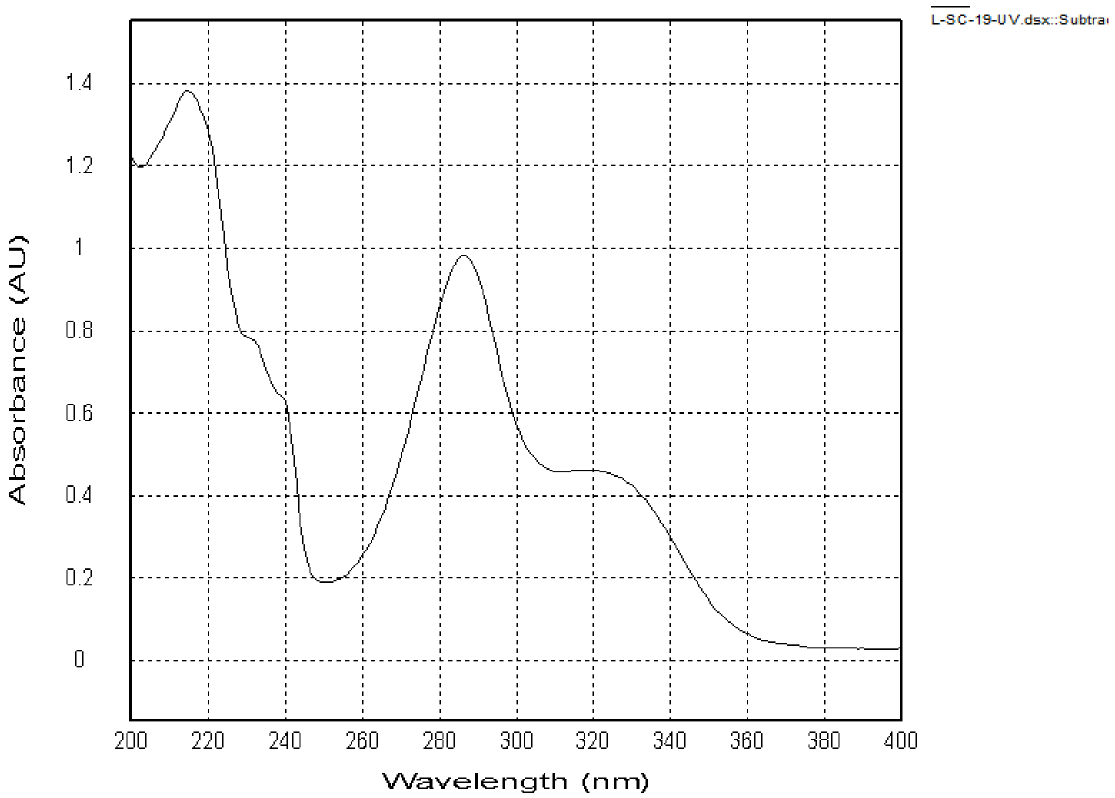

Figure S24. The UV spectrum of compound 3 in DMSO-*d*6.

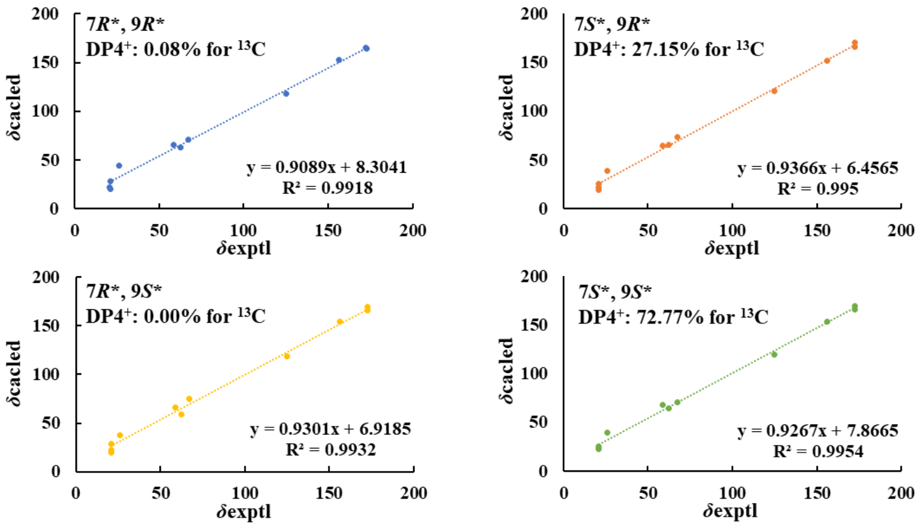

Figure S25. Linear correlation plots of calculated-experimental ^{13}C NMR chemical shift values with DP4+ analyses for potential configurations of compound 2 (Isomer 1: (7*R, 9*R**)-2, Isomer 2: (7*R**, 9*S**)-2, Isomer 3: (7*S**, 9*R**)-2 and Isomer 4: (7*S**, 9*S**)-2).**

Table S1. DP4+ analysis of experimental and calculated NMR chemical shifts of Isomer 1: (7*R, 9*R**)-2, Isomer 2: (7*R**, 9*S**)-2, Isomer 3: (7*S**, 9*R**)-2 and Isomer 4: (7*S**, 9*S**)-2.**

Functional		Solvent?		Basis Set		Type of Data	
B3LYP		PCM		6-31+G(d, p)		Scaled Shifts	
		DP4+	–	–	–	–	–
Nuclei	sp2?	Experimenta	Isomer 1	Isomer 2	Isomer 3	Isomer 4	Isomer 5
C	x	172.64	171.8	174.8	174.3	174.7	
C	x	156.19	159.1	155.6	158.1	157.2	
C	x	125	120.5	122.0	119.7	120.9	
C	x	172.43	172.6	170.2	171.0	170.7	
C		58.67	62.6	62.2	64.0	64.9	
C		62.49	59.6	63.1	56.3	60.9	
C		26.05	39.0	34.2	33.3	34.3	
C		20.88	21.4	20.1	16.2	17.4	
C		20.88	13.3	16.6	23.6	19.1	
C		67.26	69.2	71.5	73.0	67.9	
C		20.93	14.4	13.16	13.9	15.5	

Functional	Solvent?		Basis Set		Type of Data	
B3LYP	PCM		6-31+G(d, p)		Scaled Shifts	
	Isomer 1	Isomer 2	Isomer 3	Isomer 4	Isomer 5	Isomer 6
sDP4+ (H data)	–	–	–	–	–	–
sDP4+ (C data)	0.08%	27.15%	0.00%	72.77%	–	–
sDP4+ (all data)	0.08%	27.15%	0.00%	72.77%	–	–
uDP4+ (H data)	–	–	–	–	–	–
uDP4+ (C data)	–	–	–	–	–	–
uDP4+ (all data)	–	–	–	–	–	–
DP4+ (H data)	–	–	–	–	–	–
DP4+ (C data)	–	–	–	–	–	–
DP4+ (all data)	–	–	–	–	–	–

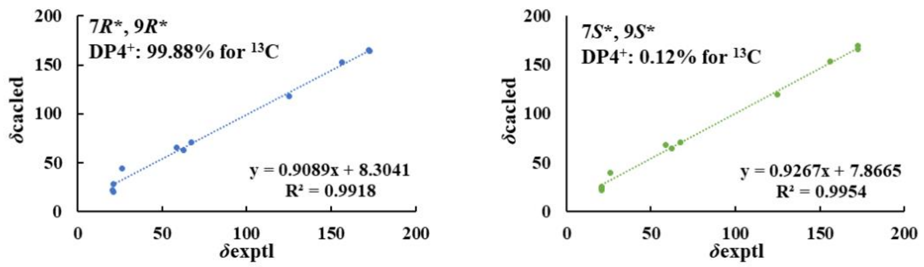

Figure S26. Linear correlation plots of calculated-experimental ^{13}C NMR chemical shift values with DP4+ analyses for potential configurations of compound 2 (Isomer 1: (7*R, 9*R**)-2 and Isomer 2: (7*S**, 9*S**)-2).**

Table S2. DP4+ analysis of experimental and calculated NMR chemical shifts of Isomer 1: (7*R, 9*R**)-2 and Isomer 2: (7*S**, 9*S**)-2.**

	A	B	C	D	E	F	G	H
1	Functional		Solvent?		Basis Set		Type of Data	
2	B3LYP		PCM		6-31+G(d, p)		Scaled Shifts	
3								
12			DP4+	–	–	–	–	–
14	Nuclei	sp2?	xperimenta	Isomer 1	Isomer 2	Isomer 3	Isomer 4	Isomer 5
15	C	x	172.64	171.8	174.7			
16	C	x	156.19	159.1	157.2			
17	C	x	125	120.5	120.9			
18	C	x	172.43	172.6	170.7			
19	C		58.67	62.6	64.9			
20	C		62.49	59.6	60.9			
21	C		26.05	39.0	34.3			
22	C		20.88	21.4	17.4			
23	C		20.88	13.3	19.1			
24	C		67.26	69.2	67.9			
25	C		20.93	14.4	15.53			

	A	B	C	D	E	F	G	H
1	Functional		Solvent?		Basis Set		Type of Data	
2	B3LYP		PCM		6-31+G(d, p)		Scaled Shifts	
3								
4			Isomer 1	Isomer 2	Isomer 3	Isomer 4	Isomer 5	Isomer 6
5	sDP4+ (H data)		–	–	–	–	–	–
6	sDP4+ (C data)		0.12%	99.88%	–	–	–	–
7	sDP4+ (all data)		0.12%	99.88%	–	–	–	–
8	uDP4+ (H data)		–	–	–	–	–	–
9	uDP4+ (C data)		–	–	–	–	–	–
10	uDP4+ (all data)		–	–	–	–	–	–
11	DP4+ (H data)		–	–	–	–	–	–
12	DP4+ (C data)		–	–	–	–	–	–
13	DP4+ (all data)		–	–	–	–	–	–

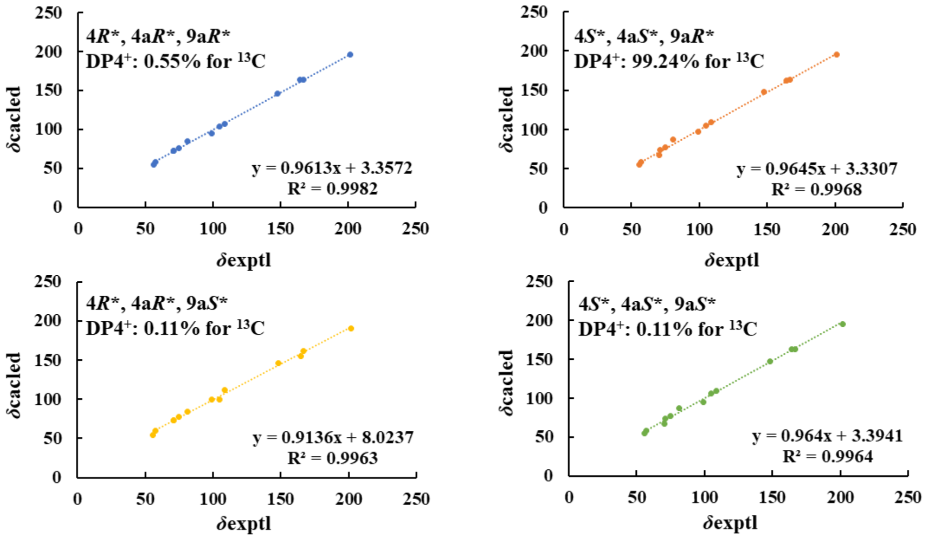

Figure S27. Linear correlation plots of calculated-experimental ^{13}C NMR chemical shift values with DP4^{+} analyses for potential configurations of compound 3.

Table S3. DP4+ analysis of experimental and calculated NMR chemical shifts of Isomer 1: (4*R**, 4a*R**, 9a*R**)-3, Isomer 2: (4*S**, 4a*S**, 9a*R**)-3, Isomer 3: (4*R**, 4a*R**, 9a*S**)-3, and Isomer 4: (4*S**, 4a*S**, 9a*S**)-3.

	A	B	C	D	E	F	G	H
1	Functional		Solvent?		Basis Set		Type of Data	
2	B3LYP		PCM		6-31+G(d, p)		Scaled Shifts	
3								
12			DP4+	–	–	–	–	–
14	Nuclei	sp2?	Experimenta	Isomer 1	Isomer 2	Isomer 3	Isomer 4	Isomer 5
15	C	x	99. 22	97. 0	95. 2	99. 8	95. 2	
16	C	x	166. 81	165. 7	167. 3	168. 4	165. 7	
17	C	x	105. 02	104. 7	104. 0	100. 3	106. 7	
18	C	x	147. 9	150. 3	147. 8	150. 8	149. 8	
19	C	x	108. 87	110. 1	108. 0	113. 4	109. 6	
20	C	x	164. 42	165. 3	166. 8	161. 0	166. 0	
21	C		71	73. 2	71. 6	71. 6	73. 1	
22	C		81. 01	86. 6	84. 8	83. 8	86. 6	
23	C		57. 34	56. 7	57. 3	57. 1	57. 1	
24	C	x	201. 5	199. 6	200. 3	200. 1	199. 5	
25	C		74. 8	76. 2	75. 56	75. 7	76. 2	
26	C		70. 71	65. 81	72. 31	71. 50	65. 85	
27	C		55. 84	53. 15	53. 47	51. 11	53. 20	

	A	B	C	D	E	F	G	H
1	Functional		Solvent?		Basis Set		Type of Data	
2	B3LYP		PCM		6-31+G(d, p)		Scaled Shifts	
3								
4			Isomer 1	Isomer 2	Isomer 3	Isomer 4	Isomer 5	Isomer 6
5	sDP4+ (H data)		–	–	–	–	–	–
6	sDP4+ (C data)		0. 55%	99. 24%	0. 11%	0. 11%	–	–
7	sDP4+ (all data)		0. 55%	99. 24%	0. 11%	0. 11%	–	–
8	uDP4+ (H data)		–	–	–	–	–	–
9	uDP4+ (C data)		–	–	–	–	–	–
10	uDP4+ (all data)		–	–	–	–	–	–
11	DP4+ (H data)		–	–	–	–	–	–
12	DP4+ (C data)		–	–	–	–	–	–
13	DP4+ (all data)		–	–	–	–	–	–

The physicochemical data of compounds 1–18.

(±)-vochysiamide C (1): colourless oil; $[\alpha]_D^{25}$ 0.0 (*c* 0.1, MeOH); UV (MeOH) λ_{max} (log *ε*): 223 (3.94), 278 (1.71) nm; IR (film) ν_{max} 2968, 2363, 1701, 1636, 1398, 1018, 1013, 698, 528 cm^{-1}; ^{1}H NMR (700 MHz, DMSO-d_6) δ_H 6.53 (d, *J* = 1.6 Hz, 1H, H-3), 4.79 (t, *J* = 5.8 Hz, 1H, OH), 3.99 (tt, *J* = 8.7, 5.8 Hz, 1H, H-7), 3.63 (m, 2H, H-9), 3.56 (dt, *J* = 11.3, 5.8 Hz, 2H, H-8), 2.71 (m, 1H, H-10), 1.15 (d, *J* = 6.8 Hz, 6H, H-11, 12); ^{13}C NMR (175 MHz, DMSO-d_6) δ_C 171.4 (C-5), 171.2 (C-2), 154.4 (C-4), 124.9 (C-3), 58.3 (C-8, 9), 56.0 (C-7), 25.1 (C-10), 20.6 (C-11, 12). HRESIMS *m/z* 214.1077 [M+H]$^+$ (calcd for $C_{10}H_{16}NO_4$, 214.1074).

(+)-vochysiamide B (2): brown oil; $[\alpha]_D^{25}$ 12.38 (*c* 0.1, MeOH); UV (MeOH) λ_{max} (log *ε*): 211 (3.01), 222 (3.12) nm; ECD (0.3 mg/mL, MeOH) λ_{max} (Δ *ε*): 209 (+0.12), 221 (−1.20), 228 (+0.93); IR (film) ν_{max} 3347, 2943, 2835, 1701, 1659, 1449, 1429, 1117, 1020, 668, 573 cm^{-1}; ^{1}H NMR (500 MHz, $CDCl_3$) δ_H 6.30 (d, *J* = 1.6 Hz, 1H, H-3), 4.24 (p, *J* = 6.2 Hz, 1H, H-9), 4.07 (m, 1H, H-7), 3.97 (dd, *J* = 12.1, 6.2 Hz, 1H, H-8b), 3.91 (dd, *J* = 12.1, 4.3 Hz, 1H, H-8a), 2.85 (m, 1H, H-11), 1.24 (d, *J* = 6.9 Hz, 6H, H-12, 13), 1.19 (d, *J* = 6.4 Hz, 3H, H-10); ^{13}C NMR (125 MHz, $CDCl_3$) δ_C

172.6 (C-5), 172.4 (C-2), 156.2 (C-4), 125.0 (C-3), 67.2 (C-9), 62.4 (C-8), 58.7 (C-7), 26.1 (C-11), 20.9 (C-10, 12, 13). HRESIMS *m/z* 228.1233 $[M+H]^+$ (calcd for $C_{11}H_{18}NO_4$, 228.1233).

4*S*, 3a*S*, 9a*R*-3a,9a-deoxy-3a hydroxy-1-dehydroxyarthrinone (3): brown oil; $[\alpha]_D^{25}$ −26.40 (*c* 0.1, MeOH); UV (MeOH) λ_{max} (log ε): 215 (3.09), 252 (2.22), 286 (2.94), 320 (2.61) nm; ECD (0.3 mg/mL, MeOH) λ_{max} (Δ ε): 212 (+19.10), 240 (−4.98), 252 (−0.63), 285 (-13.39), 308 (+5.97); IR (film) ν_{max} 2968, 2363, 1701, 1636, 1398, 1501, 1051, 1012, 669, 525 cm^{-1}; ^1H NMR (500 MHz, DMSO-d_6) δ_H 12.77 (s, 1H, OH-8), 6.73 (dd, *J* = 2.5, 1.3 Hz, 1H, H-5), 6.41 (d, *J* = 2.5 Hz, 1H, H-7), 6.13 (s, 1H, OH-4), 5.61 (s, 1H, OH-4a), 4.95 (s, 1H, H-4), 4.20 (dd, *J* = 8.0, 1.5 Hz, 1H, H-1a), 4.12 (dd, *J* = 8.0, 5.9 Hz, 1H, H-1b), 3.84 (s, 3H, H-10), 3.68 (d, *J* = 9.7 Hz, 1H, H-3a), 3.54 (d, *J* = 9.7 Hz, 1H, H-3b), 3.04 (dd, J = 5.8, 1.5 Hz, 1H, H-9a); ^{13}C NMR (125 MHz, DMSO-d_6) δ_C 201.5 (C-9), 166.8 (C-6), 164.4 (C-8), 147.9 (C-5a), 108.8 (C-8a), 105.0 (C-5), 99.2 (C-7), 81.0 (C-4a), 74.8 (C-3), 71.0 (C-4), 70.7 (C-1), 57.3 (C-9a), 55.8 (C-10). HRESIMS *m/z* 265.0720 $[M–H]^-$ (calcd for $C_{13}H_{13}O_6$, 265.0718).

2, 3, 6, 8-tetrahydroxy-1-methylxanthone (4): pale green solid; ^1H NMR (500 MHz, DMSO-d_6) δ_H 13.62 (s, 1H, H-8), 6.70 (s, 1H, H-4), 6.24 (d, *J* = 2.1 Hz, 1H, H-5), 6.09 (d, *J* = 2.0 Hz, 1H, H-7), 2.66 (s, 3H, H-11); ^{13}C NMR (125 MHz, DMSO-d_6) δ_C 181.8 (C-9), 164.2 (C-6), 162.9 (C-8), 156.5 (C-10a), 153.2 (C-4a), 152.0 (C-3), 141.2 (C-2), 123.9 (C-1), 110.3 (C-1a), 102.2 (C-9a), 99.8 (C-4), 97.5 (C-7), 92.8 (C-5), 13.7 (C-11).

(+)-griseofulvin (5): colourless oil; $[\alpha]_D^{25}$ 153.00 (*c* 0.1, MeOH); ^1H NMR (500 MHz, DMSO-d_6) δ_H 6.50 (s, 1H, H-5), 5.60 (s, 1H, H-3'), 4.05 (s, 3H, H-5), 3.94 (s, 3H, H-6), 3.63 (s, 3H, H-2'), 2.83 (m, 2H, H-5'), 2.35 (dd, *J* = 16.6, 4.8 Hz, 1H, H-6'), 0.80 (d, *J* = 6.6 Hz, 3H,H-7); ^{13}C NMR (125 MHz, DMSO-d_6) δ_C 195.6 (C-4'), 191.2 (C-3), 170.3 (C-2'), 168.6 (C-7a), 164.5 (C-6), 157.6 (C-4), 104.7 (C-3a), 104.0 (C3'), 95.2 (C-7), 91.3 (C-1'), 90.1 (C-5), 57.6 (C-4), 57.1 (C2'), 56.6 (C-6), 39.8 (C-5'), 35.5 (C-6'), 13.8 (C-6).

(*R*)-(-)-5-hydroxymethylmellein (6): yellow oil; $[\alpha]_D^{25}$ -8.97 (*c* 0.1, MeOH); ^1H NMR (500 MHz, DMSO-d_6) δ_H 11.01 (s, 1H, OH-9), 7.54 (d, *J* = 8.5 Hz, 1H, H-5), 6.86 (d, *J* = 8.5 Hz, 1H, H-4), 4.75 (m, 1H, H-8), 3.15 (m, 1H, H-7a), 2.80 (m, 1H, H-7b), 1.45 (d, *J* = 6.3 Hz, 3H, H-10); ^{13}C NMR (125 MHz, DMSO-d_6) δ_C 169.7 (C-2), 160.1 (C-3), 138.2 (C-6a), 136.0 (C-5), 129.7 (C-6), 114.7 (C-4), 108.0 (C-2a), 75.5 (C-8), 60.0 (C-9), 30.2 (C-7), 20.4 (C-10).

Bungein A (7): brown solid; ^1H NMR (500 MHz, DMSO-d_6) δ_H 9.11 (s, 2H, OH), 6.98 (d, *J* = 8.4 Hz, 4H, H-2, 6, 2', 6'), 6.65 (d, *J* = 8.4 Hz, 4H, H-3, 5, 3', 5'), 3.51 (dt, *J* = 7.2, 3.7 Hz, 4H, H-8, 8'), 2.59 (t, *J* = 7.3 Hz, 4H, H-7, 7'); ^{13}C NMR (175 MHz, DMSO-d_6) δ_C 155.5 (C-1, 1'), 129.7 (C-4, 4'), 129.5 (C-3, 5, 3', 5'), 114.9 (C-2, 6, 2', 6'), 62.6 (C-8, 8'), 38.3 (C-7, 7').

3-ethylpyrazine-2,5-dipropanoic acid (8): yellow solid; ^1H NMR (500 MHz, DMSO-d_6) δ_H 8.26 (s, 1H, H-6), 2.99 (t, *J* = 7.0 Hz, 2H, H-10), 2.93 (t, *J* = 7.3 Hz, 2H, H-3), 2.79 (q, *J* = 7.5 Hz, 2H, H-13), 2.69 (d, *J* = 7.0 Hz, 1H, H-2), 2.65 (d, *J* = 7.4 Hz, 1H, H-11), 1.21 (t, *J* = 7.5 Hz, 3H, H-14); ^{13}C NMR (125 MHz, DMSO-d_6) δ_C 174.0 (C-1), 173.7 (C-12), 154.6 (C-6), 151.7 (C-4), 150.3 (C-7), 140.0 (C-9), 32.3 (C-2), 31.0 (C-11a), 29.1 (C-3), 27.6 (C-10, 26.5 (C-13), 12.0 (C-14).

(*S*)-4-hydroxy-2,3-dimethyl-4-pentyl-γ-lactone (9): colourless oil; $[\alpha]_D^{25}$ -3.90 (*c* 0.1, MeOH); ^1H NMR (500 MHz, DMSO-d_6) δ_H 7.11 (s, 1H, OH), 1.86 (d, *J* = 1.3 Hz, 3H, H-11), 1.86 (m, 1H, H-6a), 1.80 (m, 1H, H-6b), 1.70 (d, *J* = 1.4 Hz, 3H, H-12), 1.30 (m, 1H, H-7a), 1.22 (m, 4H, H-8, 9), 1.12 (m, H-7b), 0.83 (t, *J* = 6.8 Hz, 3H, H-10); ^{13}C NMR (125 MHz, DMSO-d_6) δ_C 172.1 (C-2), 159.0 (C-4), 124.0 (C-3), 107.8 (C-5), 36.1 (C-6), 31.5 (C-7), 22.8 (C-8), 22.4 (C-9), 14.4 (C-10), 11.1 (C-11), 8.6 (C-12).

(*R*)-2-hydroxy-3-phenylpropanoic acid (10): brown oil; $[\alpha]_D^{25}$ 3.27 (*c* 0.1, MeOH); ^{1}H NMR (500 MHz, DMSO-d_6) δ_H 7.20 (m, 5H, H-5, 6, 7, 8, 9), 4.24 (ddd, *J* = 8.1, 6.1, 4.9 Hz, 1H, H-2), 3.60 (s, 3H, H-10), 2.94 (dd, *J* = 13.7, 5.0 Hz, 1H, H-3a), 2.81 (dd, *J* = 13.7, 8.2 Hz, 1H, H-3b); ^{13}C NMR (175 MHz, DMSO-d_6) δ_C 173.9 (C-1), 137.7 (C-4), 129.3 (C-6, 8), 128.0 (C-4, 5), 126.2 (C-7), 71.2 (C-2), 51.4 (C-10), 40.1 (C-3).

1-phenylbutane-2,3-diol (11): brown oil; $[\alpha]_D^{25}$ -1.83 (*c* 0.1, MeOH); ^{1}H NMR (500 MHz, DMSO-d_6) δ_H 7.26 (m, 2H, H-3, 5), 7.23 (m, 2H, H-2, 6), 7.16 (m, 1H, H-1), 4.40 (m, 2H, OH-8, 9), 3.50 (m, 1H, H-9), 3.38 (m, 1H, H-8), 2.77 (dd, *J* = 13.6, 3.8 Hz, 1H, H-7a), 2.49 (m, 1H, H-7b), 1.06 (d, *J* = 6.3 Hz, 3H); ^{13}C NMR (125 MHz, DMSO-d_6) δ_C 140.4 (C-4), 129.3 (C-3, 5), 127.9 (C-2, 6), 125.5 (C-1), 75.5 (C-8), 68.6 (C-9), 38.3 (C-7), 18.7 (C-10).

p-hydroxybenzaldehyde (12): purple grey solid; ^{1}H NMR (500 MHz, DMSO-d_6) δ_H 9.80 (s, 1H, H-1, 7.77 (d, *J* = 8.6 Hz, 2H, H-2, 6), 6.94 (d, *J* = 8.6 Hz, 2H, H-3, 5); ^{13}C NMR (125 MHz, DMSO-d_6) δ_C 190.9 (C-1), 163.4 (C-5), 132.1 (C-2), 128.4 (C-3, 7), 115.9 (C-4, 6).

4-methoxyphenylacetic acid (13): colourless oil; ^{1}H NMR (700 MHz, DMSO-d_6) δ_H 7.03 (d, *J* = 8.5 Hz, 2H, H-2, 6), 6.69 (d, *J* = 8.4 Hz, 2H, H-3, 5), 3.59 (s, 3H, H-9), 3.52 (s, 2H, H-7); ^{13}C NMR (175 MHz, DMSO-d_6) δ_C 172.1 (C-8), 156.3 (C-4), 130.3 (C-1), 124.4 (C-2, 6), 115.1 (C-3, 5), 51.6 (C-9), 39.9 (C-7).

4-hydroxyacetophenone (14): white solid; ^{1}H NMR (700 MHz, DMSO-d_6) δ_H 7.82 (d, *J* = 8.7 Hz, 2H, H-4, 6), 6.83 (d, *J* = 8.7 Hz, 2H, H-3, 7), 2.47 (s, 3H, H-8); ^{13}C NMR (175 MHz, DMSO-d_6) δ_C 196.0 ($COCH_3$), 162.3 (C-2), 130.7 (C-4, 6), 128.4 (C-5), 115.2 (C-3, 7), 26.3 (C-8).

4-hydroxy phenethyl acetate (15): colourless oil; ^{1}H NMR (500 MHz, DMSO-d_6) δ_H 9.21 (s, 1H, OH), 7.02 (d, *J* = 8.4 Hz, 2H, H-2, 6), 6.68 (d, *J* = 8.4 Hz, 2H, H-3, 5), 4.12 (t, *J* = 7.1 Hz, 2H, H-8), 2.75 (t, *J* = 7.1 Hz, 2H, H-7), 1.97 (s, 3H, H-10); ^{13}C NMR (125 MHz, DMSO-d_6) δ_C 170.3 (C-9), 155.9 (C-4), 129.7 (C-2, 6), 127.8 (C-1), 115.2 (C-3, 5), 64.7 (C-8), 33.5 (C-7), 20.7 (C-10).

Methyl 2-hydroxy-3-(4'-hydroxy)-phenyl propionate (16): brown oil; $[\alpha]_D^{25}$ 0.40 (c 0.1, MeOH); ^{1}H NMR (700 MHz, DMSO-d_6) δ_H 9.17 (s, 1H, OH-7), 6.97 (d, *J* = 8.4 Hz, 2H, H-5, 9), 6.64 (d, *J* = 8.4 Hz, 2H, H-6, 8), 5.47 (d, *J* = 6.2 Hz, 1H, OH-2), 4.14 (dt, *J* = 7.8, 5.5 Hz, 1H, H-2), 3.59 (s, 3H, H-10), 2.81 (dd, *J* = 13.8, 5.2 Hz, 1H, H-3a), 2.70 (dd, *J* = 13.8, 7.9 Hz, 1H, H-3b); ^{13}C NMR (175 MHz, DMSO-d_6) δ_C 174.03 (C-1), 155.8 (C-7), 130.2 (C-5, 9), 127.6 (C-4), 114.8 (C-6, 8), 71.6 (C-2), 51.3 (C-10), 40.0 (C-3).

Protocatechoic acid (17): ^{1}H NMR (500 MHz, DMSO-d_6) δ_H 7.33 (s, 1H, H-2), 7.28 (d, *J* = 7.8 Hz, 1H, H-6), 6.77 (d, *J* = 8.2 Hz, 1H, H-5); ^{13}C NMR (125 MHz, DMSO-d_6) δ_C 167.3 (C-1), 150.0 (C-5), 144.9 (C-4), 121.9 (C-7), 121.7 (C-2), 116.6 (C-3), 115.1 (C-6).

Apocynin (18): white solid; ^{1}H NMR (500 MHz, DMSO-d_6) δ_H 7.50 (dd, *J* = 8.3, 2.0 Hz, 1H, H-7), 7.43 (d, *J* = 2.0 Hz, 1H, H-3), 6.86 (d, *J* = 8.2 Hz, 1H, H-4), 3.82 (s, 3H, H-9), 2.48 (s, 3H, H-8); ^{13}C NMR (125 MHz, DMSO-d_6) δ_C 196.06 (C-1), 151.8 (C-5), 147.5 (C-4), 128.8 (C-2), 123.4 (C-7), 114.9 (C-6), 111.1 (C-3), 55.6 (C-9), 26.2 (C-8).
